# Analysis of Virion Structural Components Reveals Vestiges of the Ancestral Ichnovirus Genome

**DOI:** 10.1371/journal.ppat.1000923

**Published:** 2010-05-27

**Authors:** Anne-Nathalie Volkoff, Véronique Jouan, Serge Urbach, Sylvie Samain, Max Bergoin, Patrick Wincker, Edith Demettre, François Cousserans, Bertille Provost, Fasseli Coulibaly, Fabrice Legeai, Catherine Béliveau, Michel Cusson, Gabor Gyapay, Jean-Michel Drezen

**Affiliations:** 1 UMR 1231 INRA - Université Montpellier 2, Biologie Intégrative et Virologie des Insectes, Place Eugène Bataillon, Montpellier, France; 2 Institut de Génomique Fonctionnelle, Plate-forme Protéomique, CNRS UMR 5203, INSERM U661, Université Montpellier 1, Université Montpellier 2, Montpellier, France; 3 CEA-GENOSCOPE-Centre National de Séquençage, Evry, France; 4 Structural Virology Group, Department of Biochemistry & Molecular Biology, Monash University, Clayton, Victoria, Australia; 5 INRA UMR Bio3P IRISA Equipe Symbiose IRISA-INRIA, Campus de Beaulieu, Rennes, France; 6 Laurentian Forestry Centre, Natural Resources Canada, Québec, Canada; 7 CNRS UMR 6035, Institut de Recherche sur la Biologie de l'Insecte, UFR des Sciences et Techniques, Université François Rabelais, Parc Grandmont, Tours, France; University of Queensland, Australia

## Abstract

Many thousands of endoparasitic wasp species are known to inject polydnavirus (PDV) particles into their caterpillar host during oviposition, causing immune and developmental dysfunctions that benefit the wasp larva. PDVs associated with braconid and ichneumonid wasps, bracoviruses and ichnoviruses respectively, both deliver multiple circular dsDNA molecules to the caterpillar. These molecules contain virulence genes but lack core genes typically involved in particle production. This is not completely unexpected given that no PDV replication takes place in the caterpillar. Particle production is confined to the wasp ovary where viral DNAs are generated from proviral copies maintained within the wasp genome. We recently showed that the genes involved in bracovirus particle production reside within the wasp genome and are related to nudiviruses. In the present work we characterized genes involved in ichnovirus particle production by analyzing the components of purified *Hyposoter didymator* Ichnovirus particles by LC-MS/MS and studying their organization in the wasp genome. Their products are conserved among ichnovirus-associated wasps and constitute a specific set of proteins in the virosphere. Strikingly, these genes are clustered in specialized regions of the wasp genome which are amplified along with proviral DNA during virus particle replication, but are not packaged in the particles. Clearly our results show that ichnoviruses and bracoviruses particles originated from different viral entities, thus providing an example of convergent evolution where two groups of wasps have independently domesticated viruses to deliver genes into their hosts.

## Author Summary

The polydnaviruses (PDVs) are a unique virus type used by an organism (a parasitic wasp) to manipulate the physiology of another organism (a lepidopteran host) in order to ensure successful parasitism. The evolutionary origin of these unusual viruses, found in ∼17,500 braconid wasps (Bracoviruses) and ∼15,000 ichneumonid wasps (Ichnoviruses), has been a major question for the last decade. We thus undertook an exclusive work aiming at investigating this origin via the characterization of genes encoding structural components for both types of PDVs. The present paper constitutes the first report on the identity and genome organisation of the viral machinery producing Ichnovirus virions. Our results strongly suggest that Ichnoviruses originated from a virus belonging to a group as yet uncharacterized that integrated its genome into that of an ichneumonid wasp ancestor. More importantly, our results demonstrate that the ancestor of Ichnoviruses differs from that of Bracoviruses, which originated from a nudivirus. We have now identified, for the two types of PDVs, the non packaged viral genes and their products involved in producing particles injected into the host during oviposition. Together, these data provide an example of convergent evolution where different groups of wasps have independently domesticated viruses to deliver genes into their hosts.

## Introduction

Polydnaviruses (PDVs) are unique viruses symbiotically associated with endoparasitic wasps belonging to the families Braconidae and Ichneumonidae. Virus particles produced in the ovaries [1] are injected into lepidopteran hosts during wasp oviposition. The PDV genomes packaged in the particles are composed of circular dsDNA molecules that harbor from 60 to 200 genes [2]–[5]. These genes are expressed in infected caterpillar tissues, and their products ensure successful parasitism by abolishing host immune responses and/or altering host larval development [6]–[9].

Viruses belonging to a given family typically share a set of conserved genes (core genes) involved in DNA replication, transcription of viral genes and particle morphogenesis. Strikingly, PDV genomes packaged in the particles lack such typical virus genes. This is not completely unexpected given that no PDV replication takes place in the caterpillar; rather, replication is confined to the wasp ovary where viral DNAs destined for packaging are generated from proviral copies maintained within the wasp genome [10], [11]. Thus the genes involved in particle replication are not required within the particles. We recently identified genes encoding structural components of PDVs associated with braconid wasps (Bracoviruses or BVs). These genes derive from an ancestral nudivirus (nudiviruses are a sister group of baculoviruses), but instead of being packaged in BV virions they are transcribed from the wasp genome [12], [13]. Overall the data support the hypothesis that a nudivirus integrated its own genome into that of the ancestor of bracovirus-associated wasps, which lived ∼100 million years ago, according to a recent estimation based on the age of fossils in amber [14]. Since their integration into the wasp genome, the original nudivirus genes that were not essential to the parasitoid host interaction appear to have been replaced, in the packaged BV genome, by genes contributing to the success of parasitism. The nudivirus-like genes are specifically expressed in the calyx region of the wasp ovaries, where BV virions are produced, and they have been maintained in the ancestor wasp lineage and selected for their contribution to the success of parasitism as gene transfer agents for ∼100 million years.

PDVs associated with ichneumonid wasps (Ichnoviruses or IVs) share many features with BVs: they deliver genes in the parasitized host that are necessary for the successful development of the parasite, their packaged genome is composed of dsDNA circles and their virions are specifically produced in the calyx. However, IVs do not appear to derive from a nudivirus and their origin remains unclear. Indeed, the analysis of cDNAs from the wasp *Hyposoter didymator* did not lead to the identification of nudivirus genes expressed in the ovaries [12]. Moreover no set of genes having significant similarities with known viral genes could be identified. The recent discovery of a new lineage of insect viruses [15], [16], sharing few genes with other viruses, indicates that the extent of diversity of insect viruses is not completely known. This could explain our inability to identify core genes of viral origin in ichneumonid genomes. To overcome this problem, we searched for genes involved in IV particle production by analyzing the protein components of purified *H. didymator* ichnovirus (HdIV) particles and we studied the protein-coding DNA organization in the wasp genome.

IV particles have an ovocylindrical shape and a large nucleocapsid (330×85 nm) surrounded by two envelopes [17], [18]. Whereas the inner envelope is acquired *de novo* in the nucleus of calyx cells, where the particles are produced, the outer envelope is acquired from the cell membrane, during particle exocytosis into the oviduct lumen [1]. As expected from this complex structure, purified particles have a protein profile comprising several dozens of components [19], [20]. So far, only two structural proteins associated with the IV virions of the wasp *Campoletis sonorensis* (CsIV) have been characterized: p12 (gi|4101554) and p44 (*p53* gene product, gi|4101552) [21], [22]. They display no overall resemblance to known proteins, although some similarities based on secondary structure analyses between domains of p44 and of an ascovirus protein have recently been described [23].

Here we report the characterization of 40 genes involved in HdIV virus particle production. Strikingly they are densely clustered in specialized regions of the wasp genome that we named “Ichnovirus Structural Protein Encoding Regions (IVSPERs)”. These genes are specifically expressed in the calyx of *H. didymator* and at least 19 of them encode components of virus particles, including homologues of the *p12* and *p53* genes originally identified in *C. sonorensis*. Furthermore, we showed that 11 homologues of HdIV IVSPER genes are expressed in the ovaries of the ichneumonid wasp *Tranosema rostrale*, indicating that the set of structural genes is conserved among wasps associated with IVs.

Unexpectedly, IVSPERs are amplified during virus replication in *H. didymator* ovaries despite not being packaged in the particles. Altogether, IVSPER genomic structure, replication properties and involvement in particle production suggest they originated from a common set of genes, which could correspond to the genome of an ancestral virus.

## Results

### Putative HdIV structural protein genes identified amongst wasp ovarian transcripts

In order to identify genes expressed during HdIV virus particle production, 5636 clones from *H. didymator* ovary cDNA libraries were sequenced, resulting in the identification of 1956 non redundant coding regions. No significant similarities were detected with genes of conventional viruses, but two genes similar to CsIV *p12* (named *H. didymator p12-1* and *p12-2*) and two similar to CsIV *p53* (named *p53-1* and *p53-2*) were identified. Quantitative RT-PCR (qPCR) analyses indicated that transcript levels of these four *H. didymator* genes were at least 25 times higher in the calyx, where HdIV particles are produced, than in the ovarioles of female pupae (Table S1). This strongly suggested that these genes encode structural particle components, as shown for the CsIV *p12* and *p53* proteins, but also that sequences of additional IV structural genes were likely present in the libraries.

### HdIV *p12* and *p53* genes are located within atypical regions of the wasp genome

**Figure 1:**
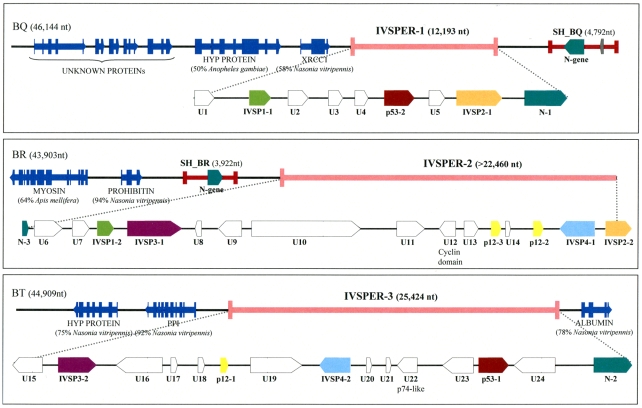
Map of the three IVSPERs identified in *H. didymator* genome and of their flanking sequences. GenBank accession numbers for BAC clones BQ, BR and BT are GQ923581, GQ923582 and GQ923583 respectively. The IVSPERs were defined as starting at the first ATG and ending at the last stop codon of the coding sequences cluster. Genes of a given family are represented by arrows of the same color. U = Unknown protein encoding gene, IVSP = gene family encoding IV Structural Protein (from IVSP1 to IVSP4). Dark blue arrows correspond to wasp genes: HYP PROTEIN = Hypothetical protein (gi|158297979| in BQ and gi|156554181 in BT); XRCC1 = DNA repair protein (gi|156554771|); Myosin (gi|156553326|); Prohibitin (gi|156538068|); PPI = phosphoglucose isomerase (gi|156554183|); albumin (gi|156543451|). The unknown proteins in clone BQ are sequences with no significant similarity to NCBI nr database entries. Proviral HdIV segments SH-BQ and SH-BR are indicated by red lines.

Several nudivirus-like genes involved in BV particle production are clustered in the wasp genome [13]. We hypothesized previously that this cluster corresponds to a remnant of the nudivirus genome acquired by the braconid ancestor wasp. To determine whether HdIV structural genes were similarly clustered, we isolated *H. didymator* genomic DNA clones containing the *p12-2*, *p53-1* and *p53-2* genes by screening a wasp bacterial artificial chromosome (BAC) library using gene-specific probes (BAC clones BQ, BR and BT; Figure 1).

Sequencing of these clones revealed that the *p12-2*, *p53-1* and *p53-2* genes reside in genomic regions characterized by a high density of coding sequences (exon density: 62.2%), making them atypical compared to the rest of the wasp genome (exon density: 21%). There was a significant difference in the mean length of intergenic sequences between these atypical regions (638 bp) and other portions of the wasp genome (1669 bp). Moreover the 40 genes in these regions consist of a single exon while a large majority of wasp genes are predicted to contain multiple exons. The atypical regions seemed to harbor virus structural genes since, in addition to *p12-2*, *p53-1* and *p53-2*, they also contained *p12-1* and another *p12* homolog, designated *p12-3*. We therefore named them “IchnoVirus Structural Proteins Encoding Regions” (IVSPER, Figure 1). Strikingly, two IVSPERs were located respectively 3 kb upstream and 4 kb downstream the chromosomal form of an HdIV genome segment that is packaged in the particles (Figure 1). These two HdIV sequences (SH-BQ and SH-BR) did not show significant similarity to each other when compared at the nucleotide level but both contained a member of the *N-gene* family that is conserved among IVs [5]. Interestingly, an *N-gene* was also present in each of the three IVSPERs (*N-1*, *N-2* and *N-3*; Figure 1).

As *H. didymator* IVSPERs contain genes (*p12* and *N*) that are related to coding sequences known to be present in packaged CsIV or HdIV DNA, we examined the possibility that IVSPERs may be part of the packaged genome as well. PCR experiments were thus conducted using specific primers and template consisting of either wasp genomic DNA or DNA extracted from purified HdIV particles. Using HdIV particle DNA, no amplification could be obtained with several primer pairs corresponding to sequences scattered along the IVSPERs, whereas amplification products could be obtained with primers specific for the SH-BQ viral segment (Table S2). This showed that the IVSPERs are not packaged in the particles but are expressed in calyx cells at the time of virus production.

### The IVSPER genes encode structural components of HdIV particles

**Figure 2:**
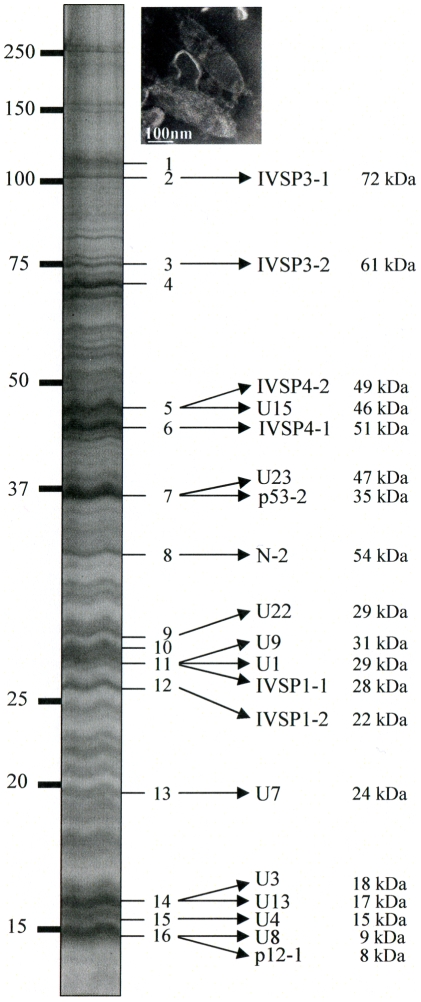
HdIV particle proteins identified by mass spectrometry and sequence searches using the translated sequences of the three IVSPER genes (16 bands were analyzed). Names and predicted molecular masses (see Table S3) of IVSPER proteins are shown beside the bands. Upper right panel shows a negative staining of HdIV virions.

**Figure 3:**
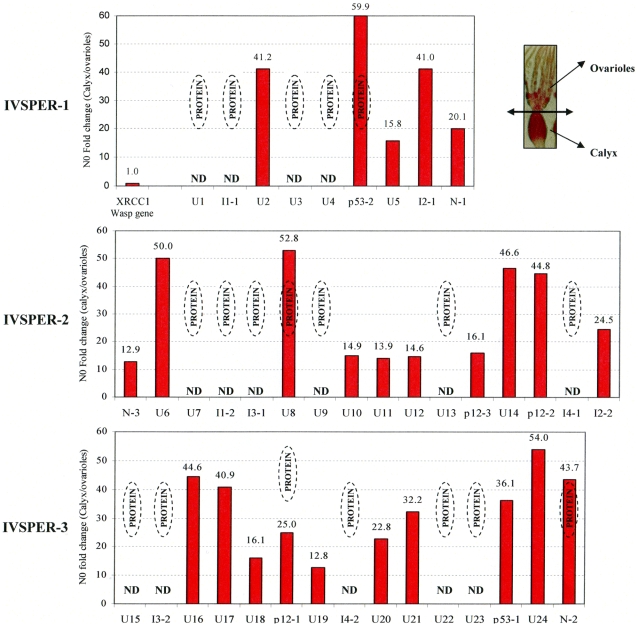
Specific IVPER gene expression in *H. didymator* calyx cells. The fold changes in transcript levels (i.e. N0 values) between calyx tissue and ovarioles are given for the IVPER genes. The calyx tissues were separated from the ovarioles under microscope as shown in the picture in the upper right panel. Amongst the genes shown by LC MS/MS to encode proteins (see Figure 2), some were not analyzed (Not Determined, ND). The wasp gene *XRCC1*, 1.2 kb upstream of IVSPER-1, was used as a negative control for transcriptional analyses. Gene names are indicated in Figure 1 (I1 to I4 correspond to IVSP1 to IVSP4).

To confirm that the identified *p12* and *p53* genes encoded structural components of HdIV particles and to assess the possibility that IVSPERs contained other structural genes, proteins extracted from purified HdIV particles were analyzed by mass spectrometry (LC MS/MS). After separation of HdIV proteins by SDS-PAGE, more than 70 bands were detected, ranging from 10 to 250 kDa (Figure 2). Among them, the 16 most intense bands were selected and trypsin digested to produce peptides. Strikingly, comparison of peptides identified by LC MS/MS with translated coding sequences showed that 19 IVSPER predicted gene products were components of virus particles (Figure 2; Table S3). They included the p53-2 and p12-1 proteins and the product of the *N-2* gene. Products of *p53-1* and of other *p12* genes were not detected, but could be present in the less intense bands (not analyzed by LC MS/MS) as other IVSPER proteins. Altogether the results obtained indicate that at least half of the IVSPER genes (19/40; Figure 3) encode virion structural proteins and that the IVSPERs constitute clusters of HdIV structural genes.

### All IVSPER genes are specifically expressed in the tissue where HdIV particles are produced

The 19 genes shown to encode components of the particles are expected to be transcribed in the tissue producing the particles, i.e., the calyx. To verify this prediction and to determine whether the other 21 IVSPER genes might also be involved in the production of virus particles we analyzed the expression of these genes by qRT-PCR. All the 25 IVSPER genes examined were found to be specifically transcribed in calyx cells at levels at least 13 times higher than in the ovarioles (Figure 3). In accordance with these results, blast similarity searches against sequence database generated from *H. didymator* ovarian cDNA libraries, using IVSPER gene sequences as queries, identified 26 different IVSPER-derived cDNAs (Table S1), thus verifying our initial prediction that cDNAs from genes involved in IV viriogenesis were present in the libraries. Altogether these results suggest that all IVSPER genes are likely to be involved in virus particle production, either directly by encoding structural proteins or indirectly by promoting their production.

### The IVSPERs encode IV-specific proteins that form seven shared families

The IVSPER gene products display no significant similarity to protein sequences deposited in public databases, and only one conserved domain has been identified in IVSPER proteins: a cyclin domain present in the U12 protein (Tables S3 and S4; Text S1). In addition, the *U22* product shows weak similarity with a baculovirus P74 envelope protein (gi|48843584|). The presence of the P74 domain was confirmed when conserved structural signatures in IVSPER products were searched for using HHPred (Table S3; Text S1). The P74 protein is an envelope protein involved in the entry of baculovirus virions into midgut cells and is conserved among nudiviruses and bracoviruses. However the presence of a single gene is not sufficient to draw conclusions as to the nature of the IV ancestor; rather, the *H. didymator* IVSPER gene products appear to constitute a set of proteins specific to IVs.

In addition to the *p12*, *p53* and *N-gene* families found in the IVSPERs, we identified members of four new gene families, named *IVSP1* to *IVSP4* (for “IchnoVirus Structural Protein”; Figure 1). Altogether members of these seven gene families represent 40% (16/40) of the IVSPER genes, and proteins within a given family display >60% sequence similarity (Table S5). The observation that IVSPERs share a combination of related genes suggests they may have originated from a common ancestor having this set of genes.

### Homologs of HdIV IVSPER genes are transcribed in the ovaries of the wasp *Tranosema rostrale*

IVs are associated with species from the Campopleginae and Banchinae subfamilies of ichneumonid wasps. The different features of the virions and the fact that PDVs have not been recorded in species from several groups separating Campopleginae and Banchinae [24], suggest that two distinct ancestral wasp-virus associations may have arisen during the diversification of ichneumonid wasps. In this context, if one assumes that the associations in Campopleginae have a common origin, the genes encoding structural proteins expressed in *H. didymator* are predicted to be conserved in wasps from this subfamily. We thus searched for IVSPER homologs by sequencing cDNAs (4992 clones) generated from the ovaries of *Tranosema rostrale* (Campopleginae), which carries the ichnovirus TrIV. As observed for *H. didymator*, no significant similarities were found with known virus genes, except with those described in IVs [4]. Strikingly, a similarity search allowed the identification of 11 genes expressed in *T. rostrale* ovaries whose products display significant similarity (60 to 93% similarity) to those of *H. didymator* IVSPERs (Table S1): seven were homologs of genes shown to encode HdIV structural proteins (*U1*, *U3*, *IVSP4-1* and *2*, *p12-1*, *U23*, *N-2*) and four to other IVSPER genes (*N-1*, *U10*, *U16*, *U19*). Interestingly these genes were not identified in the packaged genome of TrIV [4], indicating that, like HdIV IVSPER genes, they reside in the wasp genome. These results strongly suggest that HdIV IVSPER genes are conserved among campoplegine wasps and point to a common origin of the set of IV structural genes.

### IVSPERs are amplified during HdIV viriogenesis, along with proviral DNA

**Figure 4:**
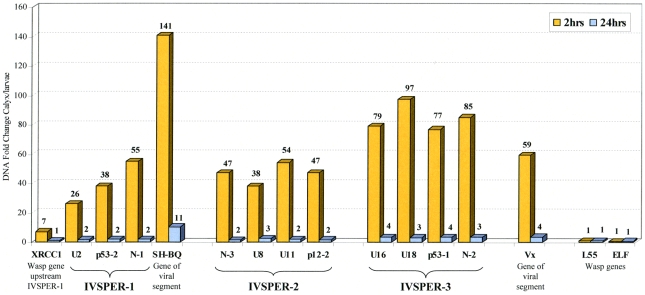
Fold change in genomic DNA amount between calyx tissue and *H. didymator* larvae for a subset of IVSPERs sequences. Results are given for calyx cells dissected 2 and 24 h after emergence of the adult wasps from the cocoons, as indicated. Viral sequences packaged in the particles (*N-gene*, SH-BQ; *vinnexin*, Vx) and wasp genes (*XRCC1* located 1.2 kb upstream of IVSPER-1, ribosomal protein L55 and elongation factor ELF1) were used as positive and negative controls of DNA amplification during particle production, respectively.

Unlike the cluster of nudivirus-like genes involved in BV particle production, two IVSPERs are located in the vicinity of the integrated form of a viral DNA sequence packaged in the particles (Figure 1). This linkage could have a role in the coordinated expression of genes involved in IV virion production. To assess whether IVSPER DNA could be amplified with the packaged DNA we studied the level of IVSPER DNA during particle production using qPCR. The levels of nine genes chosen in the three IVSPERs and of packaged DNA (SH-BQ, Vinnexin gene) were measured in calyx cells from wasps just after their emergence, when particle production is highest and in adult wasp (24h hours after emergence). As shown in Figure 4, the results indicated that the nine IVSPER genes examined are amplified in calyx cells at a level comparable to that of the viral DNA packaged in the particles. It is noteworthy that the IVSPER-3 genes, which do not appear linked to a packaged DNA sequence, are also amplified. Relative to the levels measured in 2 h-old females, there was a coordinated drop in the amplification of both HdIV segment and IVSPER DNA in females one day after emergence (Figure 4), further confirming the existence of a direct correlation between the level of amplification of these two groups of genes in the calyx. Altogether these results indicate that IVSPERs have retained an important property of virus DNA: they are amplified during virus particle production.

### Similarities between *H. didymator* IVSPER sequences and a CsIV viral segment suggest they derive from a common ancestor

**Figure 5:**
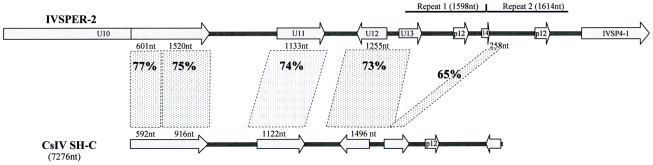
Schematic representation of the region in *H. didymator* IVSPER-2 displaying similarity to *Campoletis sonorensis* IV segment SH-C (NCBI Reference: NC_007986.1). Five related sequences, showing more than 65% nucleotide identity, are highlighted. Arrows represent the predicted coding sequences in IVSPER-2 (names as per Figure 1) and CsIV SH-C (as determined using FGENESV0 at http://linux1.softberry.com/berry.phtml). The *p12* genes are located in a less conserved region that has undergone duplication in IVSPER-2 (repeats 1 and 2).

Another link between IVSPERs and packaged viral DNAs is the phylogenetic relationship between IVSPER-2 and CsIV viral segment SH-C (Figure 5). The comparison of nucleotide sequences revealed important similarities which encompass 7014 nt in *H. didymator* IVSPER-2 and 5328 nt in CsIV SH-C. They consist in a succession of comparable (65 to 77% identity) and more divergent sequences (less than 10% similarity). The highest similarities concern regions containing coding sequences, and 5 homologs of the HdIV structural genes (including *p12* gene) are encoded, in CsIV, by a viral segment. This suggests that CsIV SH-C and *H. didymator* IVSPER-2 have a common ancestor sequence and that during evolution, the CsIV segment has retained the ability to be encapsidated whereas the HdIV segment has lost this ability and is now expressed in the calyx but not packaged.

## Discussion

### IVSPERs probably resulted from the integration of an independent genetic entity in a wasp chromosome

Because PDV packaged genomes lack typical viral genes, their relationship to conventional viruses has been a subject of debate. We recently identified genes encoding structural components of PDVs associated with braconid wasps, based on their mRNA expression in the ovaries. Present in the wasp genome and expressed specifically in the calyx, these structural protein genes resemble protein-coding genes of nudiviruses, a sister group of baculoviruses [12]. These data strongly suggest that PDVs from braconid wasps originated from a nudivirus. The same approach performed using the ovaries of *H. didymator* did not lead to the identification of coding sequences showing significant similarity to the core genes of a known virus. To overcome this problem, we conducted mass spectrometry analyses of purified virion proteins to identify genes encoding HdIV particle components.

We discovered that the proteins associated with HdIV particles are encoded by genes located in specialized regions of the wasp genome, the IVSPERs. A subset of 19 IVSPER gene products were identified as components of viral particles and the other IVSPER genes were shown to be highly expressed in the tissue where HdIV particles are produced, suggesting that IVSPER proteins contribute directly (as structural proteins) or indirectly to HdIV particle production. Thus, the IVSPERs clearly encode the protein machinery involved in HdIV viriogenesis. Consistent with this key role and the hypothesis that wasp-IV associations in this group have a common origin, IVSPER genes are conserved among IV-associated campoplegine wasps: in addition to the *p12* and *p53* genes first described in CsIV, 11 homologs of *H. didymator* IVSPER genes were found to be expressed in *T. rostrale* ovaries and four *H. didymator* IVSPER-2 genes have homologues in CsIV segment SH-C.

Analysis of the gene content of IVSPERs points to a relationship between some of the genes they contain and those packaged in virus particles, a situation that differs from that described for BVs where the packaged genome does not contain genes that are similar to those involved in particle production. More specifically, we found that IVSPERs contain members of the *N-gene* family, also present on HdIV segments and previously described in the packaged DNA of CsIV [5], *Hyposoter fugitivus* IV and TrIV [4]. The presence of related genes in IVSPER and packaged DNA, along with the absence of some CsIV genes including the *p12* gene in the packaged HdIV genome may reflect the fact that different IV genomes are at different stages of their evolution.

Except for the eight proteins encoded by the *p53*, *p12* and *N-gene* families, U12, which contains a cyclin domain, and U22, which displays a weak similarity with a baculovirus P74 protein, the other IVSPER gene products do not resemble any previously described protein. In particular, we did not find similarity with ascovirus sequences or structures, a finding that does not support the hypothesis that IVs have an ascovirus origin, as previously suggested [23]. However, the absence of conserved proteins among IV structural protein genes is not completely surprising since several sequencing programs focusing on viral genomes have led to similar findings. For example, the Mimivirus genome consists of 1262 putative open reading frames, among which only 10% exhibit significant similarity to proteins of known functions [25]. Similarly, in a comparison of the herpes virus infecting oysters and those infecting vertebrates, only the structure of the genome was found to be conserved [26].

Although they are not packaged in virus particles, IVSPERs are physically, functionally, and phylogenetically related to the packaged IV DNA and could thus be considered as an integral part of the IV genome. First, we have shown that IVSPERs are amplified in calyx cells during virus production at a level comparable to that measured for packaged segments. The genomic proximity and comparable amplification of IVSPERs and packaged segments strongly suggest they belong to common viral replication units, whereas the IVSPER-3, not in the close vicinity of an HdIV segment and flanked by wasp genes, may constitute an independent unit. A second source of evidence for a close relationship between IVSPERs and packaged IV DNA is the synteny between IVSPER-2 and CsIV segment SH-C, suggesting a common origin of these DNA regions. A simple explanation could be that during evolution of the *H. didymator* lineage, IVSPER-2 (but not the corresponding region of CsIV) may have lost the ability to be packaged. Conceptually, IVSPERs could thus be considered as elements of the IV genome that no longer require encapsidation. Due to the exclusive vertical transmission of the IV chromosomally integrated genomes, structural protein genes are not required on the viral segments injected into the host, but their amplification may have been selected for to allow production of high levels of virion structural components in the calyx. This appears to differ from the situation described for braconid wasps where the high production of structural proteins is presumed to be effected by a nudiviral RNA polymerase expressed in the calyx [13].

**Figure 6:**
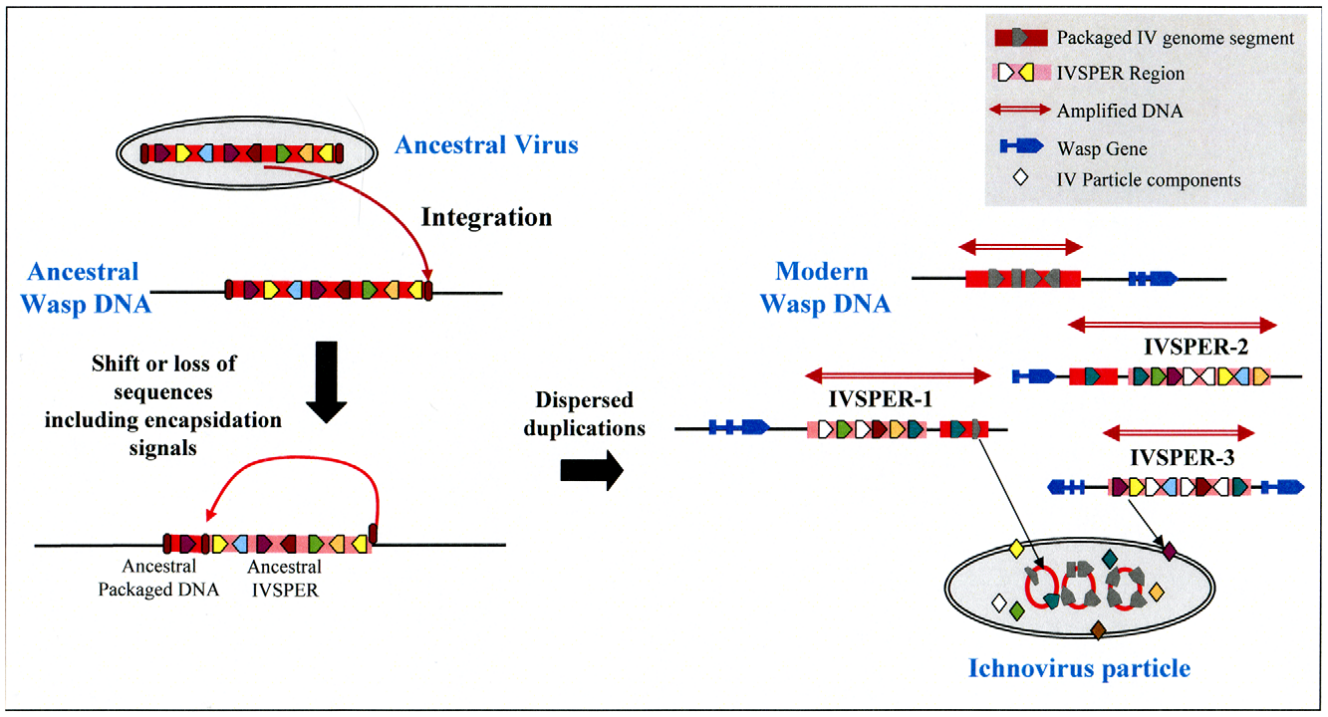
Schematic representation of a hypothetical scenario leading to the current organization of IV sequences from an unknown virus that integrated its own genome into the DNA of an ancestor wasp (ancestral virus). The loss of ability of ancestral IVSPER genes to be encapsidated may result from a shift in the position of recognition signals involved in viral DNA encapsidation (ellipses in brown red) or from other evolutive events, followed by subsequent duplications and diversification in the gene content of these DNA regions, which would have led to the modern IVs.

In addition to their functional role in particle production, IVSPERs display other notable features, including (i) their high exon density relative to regions of the wasp genome containing cellular genes, and (ii) the simple structure of their genes (made of a single exon), which is more typical of virus genes than of wasp genes, which more often consist of multiple exons. Strikingly, this organization resembles that of the “nudivirus cluster” in the genome of the wasp *Cotesia congregata*, which is thought to constitute a remnant of the ancestral nudivirus genome integrated into the genome of the ancestor of BV-associated wasps. This cluster contains 10 genes made of a single exon, is densely packed (exon density: 50%) and the products of five of its genes display similarities to conserved proteins of nudiviruses. The similar organization of IVSPERs suggests that they constitute, like the nudivirus cluster, remnants of foreign DNA integrated into the wasp genome. Altogether, IVSPER genomic structure, gene content, replication properties and involvement in particle production suggest they originated from a virus, belonging to an uncharacterized or extinct group. The nature of the ancestral virus genome could not be established using viral sequences currently available in public databases: sequences of the ancestor group are missing or IV sequences have diverged to such an extent that a relationship is undetectable. However it is interesting to note that IVSPERs contain a combination of related genes that are members of seven families. We hypothesize (Figure 6) that the IV ancestor possessed a member of each gene family. After duplications, different copies of this ancestral genome may have diversified, leading to the current IVSPERs, containing both common and specific genes that cooperate to produce HdIV particles.

Clearly IVs associated to campoplegine wasps originate from an entity that differs from that of the nudiviral BV ancestor, demonstrating that the association between wasps and viruses arose at least twice during the evolution of parasitic wasps. The use of PDVs by two groups of wasps to deliver genes into the host thus represents an example of convergent evolution. Recently a PDV from a banchine wasp has been described and was proposed to belong to a third group, based on its unusual features, in particular the morphology of the particles and the content of its packaged genome [3]. It will be of interest to determine whether this association constitutes a third event of viral capture by parasitoid wasps. Given that these associations between viruses and eukaryotic organisms have only been described for parasitic wasps, one may ask whether they are specific to these insects because of their unusual life-style, i.e. larvae living within the body of a caterpillar, or whether they occur more commonly. One might predict that virus domestication allowing gene transfer has arisen several times in the course of evolution in situations where interactions between organisms are both intimate and antagonistic.

## Materials and Methods

### Insect rearing

*Hyposoter didymator* wasps were reared in laboratory and *Tranosema rostrale* wasps were obtained from the field as described [1], [6].

### Construction of *H. didymator* ovary cDNA libraries and sequence analysis

The libraries were constructed as described [12]. Briefly, ovaries were dissected from *H. didymator* pupae of different developmental stages and total RNA was extracted using the Qiagen RNeasy Mini Kit. The cDNA synthesis was performed using the Creator SMART cDNA Library Construction Kit (Clontech) from 2 µg of total RNA. The cDNAs were cloned into the pDNR-LIB vector (Clontech). A total of 5636 clones were sequenced from the 5′-end. The sequences cleaned from vector stretches were subjected to clustering using the TIGR software TGI Clustering tool (TGICL), as described [27]. They corresponded to 597 clusters (containing more than one sequence) and 1359 singletons, and thus to 1956 non redundant sequences. To identify similarities with known proteins, the sequences were searched using the Blastx algorithm against a local non-redundant protein database (NCBI, release july 15, 2008) with no cut-off for the E-value.

### Construction of *T. rostrale* ovary cDNA libraries

Ovaries were dissected from adult wasps shortly after emergence, and total RNA was extracted using the RNeasy Mini Kit (Qiagen). 250 ng of RNA was treated with amplification-grade DNAse I (Invitrogen) [28] and reverse transcribed using an oligo dT primer, followed by a second strand synthesis and ligation of an adapter. Using a distal adapter primer and the oligo dT primer, the cDNAs were amplified and then ligated into the pGEM-T-Easy vector (Promega). A total of 4992 colonies were selected and sequenced from both ends at the Genome Sciences Centre, BC Cancer Agency (Vancouver, Canada).

### *H. didymator* genomic library construction and analysis

To obtain a *H. didymator* BAC library, high molecular weight DNA was extracted from larval nuclei and partially digested with *Hin*dIII. The fragments thus obtained were ligated into the pBeloBAC11 vector. High-density filters (18,432 clones spotted twice on nylon membranes) were screened using specific 35-mer oligonucleotides. Positive BAC clones were analyzed by fingerprint. One genomic clone was selected for each probe and sequenced by a shotgun method. Coding sequences were predicted using Kaikogas (http://kaikogaas.dna.affrc.go.jp/). A Blastn similarity search against the ovary EST libraries was performed with no cut-off for the E-value. The sizes of the intergenic regions within the IVSPER and other available genomic regions (over 1.40 Mb of wasp genome) were compared using a Student T-test (t = 4.552, df = 49.238, p-value = 3.497e-05).

### cDNA synthesis and qPCR

Total RNA from *H. didymator* calyx and ovariole fractions was extracted using the Qiagen RNeasy Mini Kit and treated with the Turbo DNAse kit (Ambion). First strand cDNA was synthesized from 3 to 5 µg of RNA using the Invitrogen Superscript III Reverse Transcriptase. Absence of DNA contamination and first-strand cDNA synthesis were verified by PCR with primers specific to Elongation Factor EF1-α (Table S6). The qPCR was performed using the Applied Biosystem 7000 sequence detection system in 96-wells PCR plates (ABgene) that comprised triplicates of 2 or 3 biological replicates. Primer pairs (Table S6) were designed using the Primer ExpressTM software (Applied Biosystems) to generate 51 bp amplicons. The final qPCR reaction volume of 25 µl contained an amount of cDNA equivalent to 20 ng of total RNA, 0.4 µM of primer pairs, and the Platinum SYBR Green qPCR SuperMix-UDG with ROX (Invitrogen). The dissociation curve method was applied to ensure the presence of a single specific PCR product.

### Quantitative data analysis

The data were analyzed either with the classical CT method or with an alternative assumption-free method [29]. The latter gives the relative N0 values corresponding to the initial transcript levels of each gene in a given tissue. Four endogenous reference genes (EF1-α, ribosomal L55, cytochrome VIIC and histone H1) were used for normalization.

### Gradient purification of virions

A first purification was performed by filtration from 300 dissected ovaries as described [30], and the viral particles were further purified on a sucrose gradient (20–50%). Centrifugation was performed at 154,324 g during 1.5 h at 4°C in a Beckman L7 ultracentrifuge, using a SW-41 swing-out rotor. Viral fractions were collected, diluted in saline buffer (PBS) and submitted to a second centrifugation (154,324 g during 1 h at 4°C) in order to pellet the viral particles. The resulting pellet was re-suspended in PBS and submitted to dialysis during two days at 4°C. The presence of viral particles was verified by TEM followed by SDS-PAGE.

### SDS-Page and protein identification

Gel electrophoresis was carried out as described [31] on a 12% acrylamide gel. After gel staining with colloidal blue (Fermentas), gel slices were cut out, washed with 50% acetonitrile, 50 mM NH_4_HCO_3_ and incubated overnight at 25°C (with shaking) with 15 ng/µl trypsin (Gold) in 100 mM NH_4_HCO_3_. The tryptic fragments were extracted with 1.4% (v/v) formic acid. Samples were analyzed online using a nanoESI LTQ-OrbitrapXL mass spectrometer (Thermo Fisher Scientific) coupled with an Ultimate 3000 HPLC (Dionex). Details are given in Text S1. Data were acquired using Xcalibur software (v 2.0.7, Thermo Fisher Scientific). Identification of proteins was performed using the Mascot v 2.2 algorithm (Matrix Science Inc.), by searching against the entries of *H. didymator* sequences. The data submission was performed using ProteomeDiscoverer v 1.0 (Thermo Fisher Scientific). Peptides with scores greater than the identity score (p<0.05) were considered as significant. All spectra were manually validated for proteins identified with less than three different peptides.

### Amplification of selected genes from wasp and viral DNA

Presence of selected genes in the HdIV packaged genome was verified by PCR using gene-specific primers (Table S6). Templates consisted of either 20 ng of viral DNA or 100 ng genomic *H. didymator* DNA. HdIV DNA was extracted from viral particles purified on a sucrose gradient (see above). Genomic wasp DNA was extracted with the Promega Wizard Genomic DNA Purification System. The 50 µl reactions were conducted using the GoTaq Flexi DNA Polymerase (Promega) following standard PCR protocol.

## Supporting Information

Text S1 Supplementary information to Materials and Methods.
(0.03 MB DOC)

Table S1 List and position of the predicted coding sequences identified in the IVSPERs of the three analyzed *Hyposoter didymator* genomic clones BQ, BR and BT. For each, the name of the gene and the results of BlastX similarity searches against the NCBI database are indicated. The “peptide” column indicates if the corresponding protein was identified by LC-MS/MS. The following columns give the qPCR results: the normalized N0 values obtained from calyx cells (Ca) and ovarioles (Ov) and the ratio (Ca/Ov). The number of clones matching the CDS sequences - by blastx searches - is given for the *H. didymator* (Hd) and *Tranosema rostrale* (Tr) ovarian cDNA libraries. Last column indicates the tblastn matches against the nr database at NCBI. U: Unknown protein. IVSP: member of IV Structural Protein gene family.
(0.13 MB DOC)

Table S2 PCR amplification results using primers specific to a subset of IVSPER genes and template consisting of either wasp genomic DNA (wasp) or HdIV packaged DNA (virus). Positive (“yes”) and negative (“no”) amplifications are indicated. One *N-gene* encoded by viral segment SH-BQ was used as control.
(0.04 MB DOC)

Table S3 List of the protein sequences found by mass spectrometry analyses using gradient purified HdIV virions. The name, the total and the non-redundant numbers of peptides found by LC-MS/MS are indicated as well as the number of the most probable HdIV protein band (see Figure 2). In the protein sequence, the peptides that have been identified are indicated in red. The estimated molecular weight (MW, in Da) is given for each of the proteins. Results of the bio-informatics analyses (see Text S1) of the sequences are indicated in the right columns. TM = predicted trans-membrane region.
(0.08 MB DOC)

Table S4 List of the protein sequences from *Hyposoter didymator* IVSPERs not found by mass spectrometry. The name and the protein sequence are indicated. Results of the bio-informatics analyses of the sequences are indicated in the right columns (see Text S1).
(0.07 MB DOC)

Table S5 Comparative analysis of the gene families found in *Hyposoter didymator* IVSPERs. Protein sequences were aligned 2 by 2 using the LALIGN program (http://www.ch.embnet.org/software/LALIGN_form.html). For each alignment, the overlaps vary in size, and the percentages of identity and similarity are given.
(0.06 MB DOC)

Table S6 List of the primers used for qPCR and classical PCR. For the housekeeping *Hyposoter didymator* genes, the identity of the Blastx match against the NCBI database is indicated on the right.
(0.09 MB DOC)
