# Supplementary material for: Analysis of Virion Structural Components Reveals Vestiges of the Ancestral Ichnovirus Genome
Source: PLoS Pathog. 2010 May 27;6(5):e1000923. doi: 10.1371/journal.ppat.1000923 (PMC2877734; doi:10.1371/journal.ppat.1000923)
Supplement: Text S1 — Supplementary information to Materials and Methods. (0.03 MB DOC) [file ppat.1000923.s001.doc]

**TEXT S1.** Supplementary information to Material & Methods.

**Construction of *Hyposoter didymator* ovary cDNA libraries and sequence analysis:** The libraries were constructed from ovaries that were dissected from *H. didymator* pupae of different stages (A (pigmented eyes), B (pigmented thorax), C (pigmented abdomen), D (emerging adults; HdIV particles present in oviduct)). A total of 5636 clones were sequenced (339, 349, 2609 and 2339 clones for the stages A, B, C and D, respectively).

**Construction of *Tranosema rostrale* ovary cDNA libraries:** The sequences of the different primers used for the cDNA libraries construction are:

- oligodT primer : 5’-TTTTGTACAAGC(T)_20_NN-3’

- upper adapter primer: 5’-CGACGTGGACTATCCATGAACGCAACTCTCCGACCTCT CACCGAGTACG-3’

- lower adapter primer: 5’-CGTACTCGGT-3’

- distal adapter primer: 5’-CGACGTGGACTATCCATGAACGCA-3’

**cDNA synthesis and qPCR:** Ovaries were dissected from pupae at stages C and D and separated into calyx and ovariole fractions. Two or three biological replicates were obtained, each consisting of tissues from 20-30 insects. Primers were designed using the Primer ExpressTM software (Applied Biosystems), using default settings optimized for the ABI Prism 7000 Sequence Detection System. We used the Applied Biosystem 7000 sequence detection system according to the recommended amplification scheme: 95°C, 2 min and 40 cycles of: 95°C, 15 sec; 60°C, 1 min. The dissociation curve method was applied according to the manufacturer’s protocol (60°C to 95°C).

**Quantitative data analysis:** The alternative assumption-free method gives the initial number, named N0 value, of molecules presents in each sample. It uses the Rn values (normalized reporter) and calculates the starting concentrations of mRNA. For each of the genes of interest and the endogenous reference genes, the arithmetic mean of N0 values was calculated from three technical replicates. We then calculated, for each tissue, a normalization factor (NF) from the geometric mean of the N0 arithmetic means obtained for the four endogenous reference genes (EF1-α, ribosomal L55, cytochrome VIIC and histone H1) from all replicates. The N0 arithmetic means from each biological replicate were divided by the corresponding NF to obtain the relative N0 values corresponding to the initial transcript levels of each gene in a given tissue. Then, fold changes between the normalized N0 means (initial expression levels) were calculated.

**SDS-Page and protein identification:** The tryptic fragments were extracted with 420 μL 1.4% (v/v) formic acid. The samples were then dried on a speed-vacuum and stored at -20°C. Samples (1 µl) were analyzed online using a nanoESI LTQ-OrbitrapXL mass spectrometer (Thermo Fisher Scientific) coupled with an Ultimate 3000 HPLC (Dionex). Desalting and pre-concentration of samples were performed on-line on a Pepmap® precolumn (0.3 mm x 10 mm). A gradient consisting of 0-40% B in A for 30 min, 40-80% B in A for 15 min (A = 0.1% formic acid, 2% acetonitrile in water; B = 0.1 % formic acid in acetonitrile) at 300 nl/min was used to elute peptides from the capillary (0.075 mm x 150 mm) reverse-phase column (Pepmap®, Dionex). Nano-ESI was performed with a spray voltage of 2.4 kV, a heated capillary temperature of 200°C, and a tube lens voltage of 140 V. A cycle of one full-scan mass spectrum (460–1600 m/z) at a resolution of 30,000, followed by five data-dependent MS/MS spectra was repeated continuously throughout the nanoLC separation. All MS/MS spectra were recorded using normalized collision energy (35 %, activation Q 0.25 and activation time 30 ms), an isolation window of 3 m/z.

***In silico* protein analysis:** Amino acid sequences were submitted to PSI-BLAST (Position-Specific Iterated BLAST) against “nr” databases at NCBI (<http://www.ncbi.nlm.nih.gov/>), to Homology detection & structure prediction by HMM-HMM comparison (<http://toolkit.tuebingen.mpg.de/hhpred>) and analyzed using the PHYRE (Protein Homology/analogY Recognition Engine) server (<http://www.sbg.bio.ic.ac.uk/phyre/>). A search for conserved domains was performed using the SOSUI system and InterProScan at the Expasy site (<http://www.expasy.org/tools/>). Prediction of protein subcellular localization was performed using PSORT II Prediction for eukaryotic proteins (<http://psort.ims.u-tokyo.ac.jp/form2.html>).
