# Supplementary material for: Analysis of Virion Structural Components Reveals Vestiges of the Ancestral Ichnovirus Genome
Source: PLoS Pathog. 2010 May 27;6(5):e1000923. doi: 10.1371/journal.ppat.1000923 (PMC2877734; doi:10.1371/journal.ppat.1000923)
Supplement: Table S1 — List and position of the predicted coding sequences identified in the IVSPERs of the three analyzed Hyposoter didymator genomic clones BQ, BR and BT. For each, the name of the gene and the results of BlastX similarity searches against the NCBI database are indicated. The “peptide” column indicates if the corresponding protein was identified by LC-MS/MS. The following columns give the qPCR results: the normalized N0 values obtained from calyx cells (Ca) and ovarioles (Ov) and the ratio (Ca/Ov). The number of clones matching the CDS sequences - by blastx searches - is given for the H. didymator (Hd) and Tranosema rostrale (Tr) ovarian cDNA libraries. Last column indicates the tblastn matches against the nr database at NCBI. U: Unknown protein. IVSP: member of IV Structural Protein gene family. (0.13 MB DOC) [file ppat.1000923.s002.doc]

| **Position CDS in genomic clone** | **Gene Name** | **Blastx (/NCBI)** | **Peptide** | **qPCR results** | | | **Nb clones in Hd cDNA libraries** | **Nb clones in Tr cDNA library** | **Matches with IVs found by tblastn against NCBI** |
| --- | --- | --- | --- | --- | --- | --- | --- | --- | --- |
|  |  |  |  | N0 Ca | N0 Ov | **ratio Ca/Ov** |  |  |  |
| **IVSPER-1 (12,193 nt) - Genomic clone BQ** | | | | | | | | | |
| 26045-26779 | **U1** | no significant similarity | YES | nd | nd | nd | 1 | 1 | no match |
| 27801-28562 | **IVSP1-1** | no significant similarity | YES | nd | nd | nd | 0 | 0 | no match |
| 29084-29770 | **U2** | no significant similarity |  | 12,0 | 0,3 | **41,2** | 9 | 0 | no match |
| 30408-30896 | **U3** | no significant similarity | YES | nd | nd | nd | 3 | 2 | no match |
| 31310-31705 | **U4** | no significant similarity | YES | nd | nd | nd | 1 | 0 | no match |
| 32220-33221 | **p53-2** | gi|223587707|emb|CAR31590.1| p53-like1 protein [Hyposoter didymator] (5e-31) | YES | 12,2 | 0,2 | **59,9** | 1 | 0 | no match |
| 33657-34187 | **U5** | no significant similarity |  | 101,6 | 6,4 | **15,8** | 0 | 0 | dbj|AB291140.1| Tranosema rostrale ichnovirus DNA, segment B1 (0.73) no CDS |
| 34568-36097 | **IVSP2-1** | no significant similarity |  | 742,1 | 18,1 | **41,0** | 4 | 0 | no match |
| 36825-38237 | **N-1** | gi|124270728|dbj|BAF45624.1| f2.2 [Tranosema rostrale ichnovirus] (3e-67) |  | 270,2 | 13,4 | **20,1** | 0 | 2 | no match |
|  | | | | | | | | | |
| **IVSPER-2 (22,460 nt) - Genomic clone BR** | | | | | | | | | |
| 21444-21725 | **N-3** | gi|45862538|gb|AAS79017.1| NHv1.2 protein [Campoletis sonorensis ichnovirus] (5e-08) |  | 92,8 | 7,2 | **12,9** | 0 | 0 | no match |
| 21840-22889 | **U6** | no significant similarity |  | 373,2 | 7,5 | **50,0** | 2 | 0 | no match |
| 23243-23881 | **U7** | no significant similarity | YES | nd | nd | nd | 5 | 0 | no match |
| 24155-24745 | **IVSP1-2** | no significant similarity | YES | nd | nd | nd | 0 | 0 | no match |
| 25271-27163 | **IVSP3-1** | no significant similarity | YES | nd | nd | nd | 1 | 0 | no match |
| 27789-28022 (-) | **U8** | no significant similarity | YES | 231,8 | 4,4 | **52,8** | 32 | 0 | no match |
| 28624-29448 (-) | **U9** | no significant similarity | YES | nd | nd | nd | 4 | 0 | no match |
| 29818-33885 | **U10** | no significant similarity |  | 96,1 | 6,4 | **14,9** | 0 | 3 | gb|AF362507.1| Campoletis sonorensis ichnovirus segment C (2e-121) |
| 35237-36187 | **U11** | no significant similarity |  | 99,0 | 7,1 | **13,9** | 0 | 0 | gb|AF362507.1| Campoletis sonorensis ichnovirus segment C (4e-142) |
| 36783-37394 (-) | **U12** | no significant similarity |  | 41,6 | 2,8 | **14,6** | 0 | 0 | gb|AF362507.1| Campoletis sonorensis ichnovirus segment C (5e-94) |
| 37664-38122 | **U13** | no significant similarity | YES | nd | nd | nd | 3 | 0 | gb|AF362507.1| Campoletis sonorensis ichnovirus segment C (5e-23) |
| 38698-39000 | **p12-3** | gb|AAD01200.1| p12 [Campoletis sonorensis ichnovirus] (6e-06) |  | 28,8 | 1,8 | **16,1** | 0 | 0 | gb|AF362507.1| Campoletis sonorensis ichnovirus segment C (2e-04) |
| **Position CDS in genomic clone** | **Gene Name** | **Blastx (/NCBI)** | **Peptide** | **qPCR results** | | | **Nb clones in Hd cDNA libraries** | **Nb clones in Tr cDNA library** | **Matches with IVs found by tblastn against NCBI** |
|  |  |  |  | N0 Ca | N0 Ov | **ratio Ca/Ov** |  |  |  |
| 39263-39424 | **U14** | no significant similarity |  | 134,7 | 2,9 | **46,6** | 1 | 0 | gb|AF362507.1| Campoletis sonorensis ichnovirus segment C (7e-06) |
| 40303-40617 | **p12-2** | gi|4101554|gb|AAD01200.1| p12 [Campoletis sonorensis ichnovirus] (0.034) |  | 30,4 | 0,7 | **44,8** | 1 | 0 | no match |
| 41235-42575 (-) | **IVSP4-1** | no significant similarity | YES | nd | nd | nd | 4 | 4 | no match |
| 43475-end | **IVSP2-2** | no significant similarity |  | 298,0 | 12,2 | **24,5** | 0 | 0 | no match |
|  | | | | | | | | | |
| **IVSPER-3 (25,424 nt) - Genomic clone BT** | | | | | | | | | |
| 15761-16993 (-) | **U15** | no significant similarity | YES | nd | nd | nd | 1 | 0 | no match |
| 17645-19228 | **IVSP3-2** | no significant similarity | YES | nd | nd | nd | 0 | 0 | no match |
| 20085-21923 (-) | **U16** | no significant similarity |  | 298,3 | 6,7 | **44,6** | 3 | 1 | no match |
| 22269-22517 | **U17** | no significant similarity |  | 75,4 | 1,9 | **40,9** | 24 | 0 | no match |
| 23313-23570 | **U18** | no significant similarity |  | 49,9 | 3,1 | **16,1** | 0 | 0 | no match |
| 24256-24489 | **p12-1** | gi|223587709|emb|CAR31591.1| p12-like1 protein [Hyposoter didymator] | YES | 110,4 | 4,4 | **25,0** | 227 | 6 | no match |
| 25532-27514 | **U19** | no significant similarity |  | 305,7 | 23,9 | **12,8** | 0 | 1 | no match |
| 28304-29599 (-) | **IVSP4-2** | no significant similarity | YES | nd | nd | nd | 4 | 1 | no match |
| 30221-30433 | **U20** | no significant similarity |  | 502,3 | 22,0 | **22,8** | 4 | 0 | no match |
| 31014-31253 | **U21** | no significant similarity |  | 231,8 | 7,2 | **32,2** | 1 | 0 | no match |
| 31501-32274 (-) | **U22** | no significant similarity | YES | nd | nd | nd | 0 | 0 | no match |
| 33345-34592 (-) | **U23** | no significant similarity | YES | nd | nd | nd | 12 | 5 | no match |
| 34827-36014 | **p53-1** | gi|223587707|emb|CAR31590.1| p53-like1 protein [Hyposoter didymator] |  | 27,9 | 0,8 | **36,1** | 3 | 0 | no match |
| 36278-37969 (-) | **U24** | no significant similarity |  | 147,6 | 2,7 | **54,0** | 2 | 0 | dbj|AB291140.1| Tranosema rostrale ichnovirus DNA, segment B1 (5e-04) |
| 39706-41184 | **N-2** | gi|124270728|dbj|BAF45624.1| f2.2 [Tranosema rostrale ichnovirus] (2e-89) | YES | 644,4 | 30,6 | **21,1** | 1 | 2 | no match |
|  |  |  |  | 65,1 | 1,0 | **66,3** |  |  |  |

**TABLE S1.** List and position of the predicted coding sequences identified in the IVSPERs of the three analyzed *Hyposoter didymator* genomic clones BQ, BR and BT. For each, the name of the gene and the results of BlastX similarity searches against the NCBI database are indicated. The “peptide” column indicates if the corresponding protein was identified by LC-MS/MS. The following columns give the qPCR results: the normalized N0 values obtained from calyx cells (Ca) and ovarioles (Ov) and the ratio (Ca/Ov). The number of clones matching the CDS sequences - by blastx searches - is given for the *H. didymator* (Hd) and *Tranosema rostrale* (Tr) ovarian cDNA libraries. Last column indicates the tblastn matches against the nr database at NCBI. U: Unknown protein. IVSP: member of IV Structural Protein gene family.
