# Supplementary material for: Analysis of Virion Structural Components Reveals Vestiges of the Ancestral Ichnovirus Genome
Source: PLoS Pathog. 2010 May 27;6(5):e1000923. doi: 10.1371/journal.ppat.1000923 (PMC2877734; doi:10.1371/journal.ppat.1000923)
Supplement: Table S2 — PCR amplification results using primers specific to a subset of IVSPER genes and template consisting of either wasp genomic DNA (wasp) or HdIV packaged DNA (virus). Positive (“yes”) and negative (“no”) amplifications are indicated. One N-gene encoded by viral segment SH-BQ was used as control. (0.04 MB DOC) [file ppat.1000923.s003.doc]

| **Gene name** | **PCR amplification results** | |
| --- | --- | --- |
|  | **wasp** | **virus** |
| **IVSPER-1** | | |
| **p53-2** | Yes | No |
| **N-1** | Yes | No |
| **SH-BQ N gene** | Yes | Yes |
| **IVSPER-2** | | |
| **U8** | Yes | No |
| **U9** | Yes | No |
| **U11** | Yes | No |
| **U13** | Yes | No |
| **p12-3** | Yes | No |
| **U14** | Yes | No |
| **p12-2** | Yes | No |
| **IVSP4-1** | Yes | No |
| **IVSPER-3** | | |
| **p12-1** | Yes | No |
| **p53-1** | Yes | No |
| **N-2** | Yes | No |

**TABLE S2.** PCR amplification results using primers specific to a subset of IVSPER genes and template consisting of either wasp genomic DNA (wasp) or HdIV packaged DNA (virus). Positive (“yes”) and negative (“no”) amplifications are indicated. One *N-gene* encoded by viral segment SH-BQ was used as control.
