# Supplementary material for: Analysis of Virion Structural Components Reveals Vestiges of the Ancestral Ichnovirus Genome
Source: PLoS Pathog. 2010 May 27;6(5):e1000923. doi: 10.1371/journal.ppat.1000923 (PMC2877734; doi:10.1371/journal.ppat.1000923)
Supplement: Table S3 — List of the protein sequences found by mass spectrometry analyses using gradient purified HdIV virions. The name, the total and the non-redundant numbers of peptides found by LC-MS/MS are indicated as well as the number of the most probable HdIV protein band (see Figure 2). In the protein sequence, the peptides that have been identified are indicated in red. The estimated molecular weight (MW, in Da) is given for each of the proteins. Results of the bio-informatics analyses (see Text S1) of the sequences are indicated in the right columns. TM = predicted trans-membrane region. (0.08 MB DOC) [file ppat.1000923.s004.doc]

| **Name** | **# Peptides** | **non redundant peptides** | **# most probable band** | **Protein sequence** | **Estima-ted MW** | **SOSUI prediction** | **InterPro Scan prediction** | **Psort prediction** | **Psi Blast search Evalue <1.e-2** | **HHpred search >80% probability** | **Phyre search >50% precision** |
| --- | --- | --- | --- | --- | --- | --- | --- | --- | --- | --- | --- |
| **IVSPER-1** | | | | | | | | | | | |
| **U1** | 10 | 6 | 11 | MSRYLITDEDFDLMNQVAVIQVQNEIVDSKWEYEKKANRQDFFKLSSKPWSSNIKDRLKKSARDMIPTEIKSLRLTKEGCKHYACPSIVYIKHLGEIDINKVDVASLVKDDMPDVSEYCFQKYRRRLIERDNPRMKSPFDHIGNLQWRKMSDGHMGCSQDNEIFRNWALYPWTRAKTFKDGVTNAPPLVLHDDNQFDMTEKYCRFKGVSYKKADEGKHNDDCYVSRGQAVAETILGYWPRDLYR | 28798.7 | soluble protein | no hit | 56.5 %: nuclear | HP | none | ADP-ribosylation/Hydrolase/Topoisomerase V, 50% precision, E>2.9 |
| **IVSP1-1** | 12 | 3 | 11 | MDLDFETIAKIAEAFPAADFASDGNADNQLGDSTEIQKEGNKHLRFVKMLYGLIIQAHVLSWVAAGMFILGSLSDLYLQTSYITDHIIVGIGSVLWILYAYSKHNYPVLLQNLIVGGISVYVFLRHLNMGKFAYLIAALLCMSCAVNRHENVMISTNPNIIPSQCSNISNAASNHVIAMRSNSHPPICELEVIGKVLDEKIKVIGELQQILDG VAESSGTGQIVNHNYDDMYKTARNMIDSVLKDVRSKMQKC | 27949.3 | membrane protein (4 TM) | 4 TM | 88.9 %: endoplasmic reticulum | HP | none | Topoisomerase V/virion rna polymerase bacteriophage/diphteria toxin, 80%/65%/60% precision, E 0.5/1.6/2.2 |
| **U3** | ? | 2 | 14 | MFGAVHSKISPATAALYGYQQTQWLSELIWLQSGSAHENLQFSKRGDGTTTNDPVSGDIKCVKEHGGMIVMTYDDSIDQLVPKCKCTARQVWQGPSCETPNPMYCNGGGANAIPVWENGQWQCKQQGSNSETPPETPVVLNMHNKQIFVYGDNKRQRLELLY | 18024.2 | soluble protein | no hit | 44.4 %: extracellular, including cell wall | HP | none | none |
| **U4** | ? | 4 | 15 | MKAQPRPVRRRGKSHRRKPGITAQAVGILSTTLSTAVAFVLGRQLWRTRIGEWTYDKDELLTYRKTRLSRFARGFGYEEARENHLLALLKSSITEDDNTNLLLHVLFPYLTMTDESNTAASKDAGNDASKQ | 14864.9 | soluble protein | 1 TM | 43.5 %: nuclear | HP | PIRSF000350 Hg-II_reductase_MerA. prob=95.70, Evalue=0.0054, Pvalue=5.1E-08 | none |
| **p53-2** | 133 | 13 | 7 | MSKPEHAVKSGAANEPAQVGAGGNAEDVDVATDEQLNKELESHTYPMNCVKGMRLKKIAKYPKHHDKGSPFESHIVVTEKHNKVIRVGAIIVNGRAVKGSDKISHWKGLRLNPNYMDALLTSLGESIPNSVHAFQAKLRYPDDFVLAIIAPPNITMFPDEPLPEGFVPDTSLQYEIVVQQNKHDPDRLTSAKKSSIKRWFKDFTWKSVSEKVARAEQKCSHEDKTVGYVAMPESGHTHVPIRASAPRLDDEPYGFHHRLRKRMSTDSSHSNDVTGTKKPSEGIQAAMQTYGIPAVLAIGSAGAAIGGAVWLSKSPRMKSYIGL | 35491.3 | membrane protein (1 TM) | 1 TM | 26.1 %: cytoplasmic 21.7 %: nuclear | gi|4101552|gb|AAD01199.1| **p53** [Campoletis sonorensis ichnovirus] Evalue=8e-19 | none | none |
| **IVSPER-2** | | | | | | | | | | | |
| **U7** | 2 | 2 | 13 | MSNMPHTSSKQSIDDVLGERVFLLNAPRLFEQRQQLESYATNQDEQLRLASSSAEQFFKPIKNVIPSLAQIDHQPLVLKEEYKVCNSVEKTFSRNGDKLVNGADQKAGKKTRSLWSYDNLLKILMLLLLLAIYVEVRKSDEPIVDAHSFGSFNNSFITSAVGYGSDTLMEKMSGVWQTYDSACQSAKLVYHRIRTALSLSPVIGIHATAAET | 23732.0 | membrane protein (1 TM) | 1 TM | 43.5 %: nuclear | HP | none | none |
| **IVSP1-2** | 3 | 2 | 12 | METELLAWLGAILYTAGSVSDLFLDF**VYVIDHSILAAGSILWMLYALA**KKEYPLLLQHTVMLLISMYVLIKQLNIGKFAYLTTVLLCTTCSVYRHESVMGDNLACGTASVTDTPNAIGMTSAYRRPVCDFETVYLTLDDKIEIVDQINRILIYLNGNDSEDGPLKNNLYRHIFISAETQMTQILGSLRDKKHSCQS | 21999.5 | membrane protein (3 TM) | signal-peptide and 2 TM | 34.8 %: cytoplasmic 21.7 %: mitochondrial | HP, gi|168212716|ref|ZP_02638341.1| transposase A from transposon [Clostridium perfringens CPE str.  F4969], Expect = 0.82 | none | none |
| **IVSP3-1** | 2 | 2 | 2 | MTTSLRQILASWVYAHVSNCLDNDADIEQNRVDLAVPPPGGEMHKFYTFLLKIEDILRDKSFGGRYDQWISDDLHFIVPPEDLDKPITWKYLKRLCRLLHFFNNMKNEPPLLSVLAKHVPGVVKNLDTLEKSEPTFASLLFVVQMKGKLRDTASVSNALDQLMHRILGTDDDRVNGIFDLTKKQSYVFVNGVLDLARQLFEPILQYLPAEDKESLTILDLVKERLPESLLQQTLWKNLETSAVNEVGSSDVRRLRSSKQEQGPLCWEFISALLSEKYCDDVLHDEVFEKNDLRGFHRFLDGASAHENETLQGQESAVEKKHIFKLSMYSISALLLLSSKTLVHCQCATKTVQFVSLIFATFDEAKLKMQSLANCKVLVFASHEVSKFPMALPSDRFNFRNMFITYRQADIFERPPGTIFKYEYLNELNFIRMLEIAGLQPTQETLIHLLLELKNDPTVVLSDFLYLQLLHTITYSFGSPPFTSDLDNSEIRERYRSLSSTNALLRLYGAGVYNATTIFCLSFVQKMAYFSHRAVLRAINLIPIIQFIDFNTRNNLYPITSAMLQMYLRHLPGRHNNSTALTSASVDSQHMATITMFKYLSLVVNVMILEHTKGKYTMVDFIEQSGQITLY | 72256.3 | soluble protein | signal-peptide | 34.8 %: cytoplasmic 30.4 %: extracellular, including cell wall | HP | none | E=0.00086, precision 95%/ Heat repeat d1b3ua / E=0.001, precision 95%, phage n4 rna polym. |
| **U8** | 2 | 2 | 16 | MSQTALARRGRRRSMNNSKEIDDVLLAIDDLLQSLIIKTASERSQWANILFDSVRTIQSDSTFTATDSKTGNKVLYA | 8634.7 | soluble protein | no hit | 43.5 %: mitochondrial 26.1 %: nuclear | HP | pfam00760 Cucumovirus coat protein. prob=83.90, Evalue=1.4, Pvalue=1.3E-05 | none |
| **U9** | 43 | 10 | 11 | MNILGIVSLLDKIDVSAMKTVSMIVNLNDANVLTRKLNDISKASCSLTGYIPFLCSVQISDVCIEDENGLRKTVYSLSNKTDEESEYADLVIEKIIVCIGLRKSDGISNLISKFFSSSSSNGDNDSDNLDFINHIVKEYCPEIKILTESKYCSEATVCLQLDAKSLDLRDWRRTITRVMEHWKTMFDLNRIDATTIKNSKINVLHDDGSKKSTLNDLSRLNNECIRDIELEFSYATPVSAPVKKDGDASPQRRKRRASNDVSDIACKRFADDVK | 30779.0 | soluble protein | no hit | 52.2 %: nuclear | HP, gi|118363230|ref|XP_001014873.1| Viral A-type inclusion protein repeat containing protein [Tetrahymena  thermophila SB210], Expect = 0.030 | none | E=3.2, precision 50%, phage n4 rna polym. / E=4.8, precision 40%. Dna mismatch repair msh6 /E=6.4, precision 30%, topoisomerase V |
| **U13** | 105 | 9 | 14 | MAFVSAIVKNIFLNDSKETEESNPFLDPLPIFNCPVFYARTLNLLLSPKNASQNSNLAFRPTTLFTAEGLTVPVYGKSHQPVDVIPEAFSDDLMTRPYMLQELKDNVIYGDSVLPDGFSDGDNGNTDGADASMSYMKNPTKAWTPSSIVVQP | 16697.8 | soluble protein | signal peptide | 43.5 %: nuclear 30.4 %: cytoplasmic | HP | none | none |
| **IVSP4-1** | 558 | 27 | 6 | MTPLSIIRSEECTSYDSVDVPCVTIKRPKVIRFRERTPFDISFLHKGQKVRSVGEALRIVRLVVPWTTAKPEKIREILRTLNVWNFPTPRKSPNITEIAFRFGLQHFYRYCCISEGNKLVYDGTYFDDLTDYLDESGYCNGMLVWLPPDLICDTLTFIKTFDSNIDEPVEDGKGNVDSKQAAADDLQENNFKSLFKMKSFITDSTDYESKNGIIATNYIEAKHVIVSFIKGLLAIQSQINSSVLEFFSKQWLSNIYRGFCQFTSKEAFCMFFNNVGIFQFSQHIMFYVPNAHGKFTSRLVIPEKFIPNAEEQSISNGRNSVIHGAREDPDEGISDILGRVCGFKLSDDVAISEMDTLAMKKLNRIPSMIIQMCPSSEGANSLSDGQFRTISEIFGGLGKSSHVKGSRKVGRINYDDEVDEDDDYDEDEVLSKVNVRPKLKKRVKFA | 50723.7 | soluble protein | no hit | 47.8 %: cytoplasmic | HP | 189-247:TIGR02386 rpoC_TIGR DNA-directed RNA polymerase, beta' subunit Probab=92.30 E-value=0.05 | E=0.31, precision 85%, phage n4 rna polym. |
| **IVSPER-3** | | | | | | | | | | | |
| **U15** | 2 | 2 | 5 | METIMFLASGSVALTQIIRKCLEVPGPRKIPKPTDVTPLSLSDENSESIFHARTYFNKQRETPIQPVIAIEELDTPCVTTHDCTVRFGDGFACLNLDT PVRMHITVEDVAKSVIFGGNNEKPRADDADVARSLVFGGDVKPSVKNEVKNCKRCVPVGKSRRVIKCNPLTSIAVCSSVFSPQTNRFDAHWVCESLYPEMFTEDPETGDITKCRACGADENRGRLIHEVSRLPWDKHYERHGLYDPLDSLKCECYPFPFSPEEAETKRSLYAGNILLLPPTSTCRRSNCAPGRPHMSDPQACDCPAGYVSCPILFHKNNKALHERLCWGSYVRNTRDHPMPSCVRDPCMPHAKMDPSTGTCVANDGDVTVVNDKNIFPNAPWGVPIKRKQTIICGTDMEAPLKKKKYIELKY | 45816.5 | soluble protein | signal peptide | 69.6 %: nuclear | HP | none | none |
| **IVSP3-2** | 2 | 2 | 3 | MATFQKALQKHLPLVVRELDTLEKKEPPYKSLLSALRLSGNLRNTVTVFNALDQLLHRLLGTDDSRLSSLFKLDKRQSYVYVNGVLDSARQLFEPIVQYLPAEERKHSTIIELVNERIPDSLLQETLWKNLGTTPLYEVSAADVERLQTTDQQQGSLCWEFITGLISMKYYTDILNKEVFNGKELKKLQSVLNARPDPGVKCKYNEEKENRTNFVVKRVFKLSMYSIAALLLLSSKTMLQCPCAAKTRNFISLVFSLCDEIKLQAKSLENCKLLVFAAHEISKFPMVEPADRFKFRNLFITYRQEDIFGRPTGHMFKYKYLNELNFLRQIETAGLEPTYELLLHLLLTLKNDRSVSFSDLLYFQILNAICSSFGFAPITLEWKKPEEIEKYRTLSSKSSLLRLYGKGSYDAFTIFCLAYVQKMAYFNDQPMDSIKHLLPLIHLMYFNTKNNVYPITCAFLEMYMQHLPCDHMKSLIDGSSCLESKQFGTQLLFKYLSLVVNVMMMEQTKGQYTMVDFIEQSGEITLY | 61023.0 | soluble protein | no hit | 47.8 %: cytoplasmic 30.4 %: mitochondrial | HP | none | E=7.1E-3, precision 95%, HEAT repeat, d1b3ua / E=0.026, precision 95%, bacteriophage n4 rna polym. |
| **p12-1** | 16 | 2 | 16 | MSY**LSLGSSVLGASLSVLTCYEVFQV**EVTEGNKKNLLIAKVASVASATSSLVSGGINVYDMIQSDDSSSKYLDLNAS | 8028.0 | membrane protein (2 TM) | signal peptide | 39.1 %: nuclear 26.1 %: cytoplasmic | gi|4101554|gb|AAD01200.1| **p12** [Campoletis sonorensis ichnovirus] Evalue=1e-06 | none | none |
| **IVSP4-2** | 271 | 23 | 5 | MATTAIMKTNTCLAYDTVTLPCATIKSPKMIKFSGNTTFDISFIHPGQKVQSVDEALRFIRTIVSWTTSSVDKIREILRALGTWKFPTPRRSSNIFEITYRFALQHFYRYCCISDGSKLIYDETYFDDLNDYMGENGYVNGQIMWLPPALICETLLFIKEYDENIDYQPEDDQENENYSMSDNLDGNSVNISKLPSVPQSRQDSKAIKTFAGHVVTTNYTEAKNIILSFIKEVLTVESQVNTSALQFFSKQWLSNVYKGCYQFASREAFCMFFNNICIFEFSQHVMFRVPDIRGAFTRRLIIPEVNMKNIQKPTNSKVRDSSSRRVKEGSNEEIGDVLGQICGFKLVDDAPGSEMDLKAINKLNRIPSMIVRMCPNDDDSSVGGGSQLRTATEILGGIAKGTSGRSKNSAIANHAVLPAGRRSAKTSRLRK | 48587.3 | soluble protein | no hit | 47.8 %: nuclear 34.8 %: cytoplasmic | HP | none | E=1.2, precision 70%, bacteriophage polym. |
| **U22** | 70 | 13 | 9 | MVLKKLIMEVATALESEACQAIIRQITSKIGQKLVTELGMRAVVAGVAENMAGVLAAMGPIGWVIDGLMLVFTIVAIVYNHYDPHNYSQTYFRETIENMLRDIEDSTKYETYKFAKSMNRTFDYPIELNGTDLFDEREWKYDVQKLLMNSLSYGKELVANLYRNSRGEIIDLQALCETDENIDRSLITHPPSWNTTIQRSVETVISSQERAHAIQHELEENLKLWDGLAPVGWISLLTVGCTVAFYELTKPQINSTD | 29191.3 | membrane protein (1 TM) | 2 TM | 34.8 %: cytoplasmic 21.7 %: mitochondrial 17.4 %: nuclear | HP, gi|48843584|ref|YP_025157.1| P74 enveloppe protein [Neodiprion sertifer nucleopolyhedrovirus], Evalue=0.87 | PF04583 Baculo_p74: Baculoviridae p74 conserved region; ODV envelope protein. prob=97.78, Evalue=0.0021, Pvalue=2E-08 | E=0.93, precision 75%, topoisomerase V |
| **U23** | 600 | 27 | 7 | MVGYQQTPWCSNTTMQNGEAVLSDARLIKIIHTFFELDDLKMSCNDMLTKVLSVFRDAIATGGLQEILNGFMLFLCGRREKHHGSPIPRIATYWDFKILIAGLLKRFSNDKSLKDVLDTYDDTGVVDRMVTGGYSLRLNSNIISMMDTAPYAGIRLLPPTKLLVLEPKQAAVLVYLFRTYYSNNTGIDDSAVRNFMGMLEKIDESIHGSVSARESDFRPVKSASRKTILATHQDASLVVRKYIMGQYSYIFSMLNQCFLRYDFLNNTEATHPRYQTKELVLNYLSSLMGLPANACEEHVWTTYVNDVGAVTSLTSMLDSGATNMEFLTYIVLQSLMLVQSASNEGQSYHEQYMKLRGTVAEQLRMGRYSKENPYDPGVIRKDDASLNIGPEKLKCLQALNKTIESQGPRGVSFPK | 46764.7 | soluble protein | ATP synthase a subunit signature [133-142] | 52.2 %: cytoplasmic | HP | 187-270:PRK05755 DNA polymerase I. prob=94.4, Evalue=0.07, Pvalue=6.6E-07; 175-255: pfam00747/g Viral_DNA_bp ssDNA binding protein. Probab=74.42 E-value=15 | E=0.034, precision 95%, bacteriophage n4 rna polym. |
| **N-2** | 5 | 5 | 9 | MYPHEDDDSRGSVGSPPAQGEFDHGSISECEDDLEEEYNDDAENSEAEMNQAASGDEEFDCPRTSPQPLPVAEPISPEAQPPTSMKKIAKRKPAAAKSVPVSRTGKPGHEEQAVSAGSAEKKKALGVPKKRPAVKPIAKGDSVDLAKKPSAPKKKPVSKLKDPMEKASCALPSDDPRNKTKVAVKKSRKLVAAATDKPPVKKGRQDVKHRSEEASSLQGINGMSPESMAAEVHAHSLPQVNIPRKKKTSDETSAKNPSILSSESSDRRFWMETLAGDFKSTGYNALLKNAHFTEQQANVLQQSFYQLCRKLSDISTYSDRFMKTHKCIECEFSISHQCVSIDFTQYVPSSANVTGVTTITPQSTTMCICQFGFFHSHATQPRLKLANNLSLKSTLRMLEHNRSVSVSCPRCHMNVVIKNRNSDVCCDFTNWNSLEGGNRRQFFNSLENRLQTSKMSRPYLNELYTCNRQCCQLFHRCAENAVADNTISMRPH | 54450.3 | soluble protein | no hit | 87.0 %: nuclear | gi|45862538|gb|AAS79017.1| **N**Hv1.2 protein [Campoletis sonorensis ichnovirus] Evalue=1e-83 | none | none |

**TABLE S3.** List of the protein sequences found by mass spectrometry analyses using gradient purified HdIV virions. The name, the total and the non-redundant numbers of peptides found by LC-MS/MS are indicated as well as the number of the most probable HdIV protein band (see Figure 2). In the protein sequence, the peptides that have been identified are indicated in red. The estimated molecular weight (MW, in Da) is given for each of the proteins. Results of the bio-informatics analyses (see Text S1) of the sequences are indicated in the right columns. TM= predicted trans-membrane region.
