# Supplementary material for: Analysis of Virion Structural Components Reveals Vestiges of the Ancestral Ichnovirus Genome
Source: PLoS Pathog. 2010 May 27;6(5):e1000923. doi: 10.1371/journal.ppat.1000923 (PMC2877734; doi:10.1371/journal.ppat.1000923)
Supplement: Table S4 — List of the protein sequences from Hyposoter didymator IVSPERs not found by mass spectrometry. The name and the protein sequence are indicated. Results of the bio-informatics analyses of the sequences are indicated in the right columns (see Text S1). (0.07 MB DOC) [file ppat.1000923.s005.doc]

| **Name** | **Protein sequence** | ***SOSUI*** | **InterPro Scan** | **PSORT** | **Psi Blast** | ***HHpred*** |
| --- | --- | --- | --- | --- | --- | --- |
| **IVSPER-1** | | | | | | |
| **U2** | METMDPNADTDGAASSDNNDDHTYEKLIELCHCTTLAIVAFTFQTEIELIVSDTEANSYEFSTFEDFQKFLVFVRELRAIVFHLRQNGDPAVYVSIASITVFRDLLPYSGSSMIQMLLKVHTLVEIIKSQWKLSLSEPPCKGLAHRMIRHMTNSGTLAREAAALRQDLKFVHALLAEFHLSGSNHETVTEIYQTYIDYIEDRMTALGQSSKRQSNNQQHWSQKRQRFE | soluble protein | no hit | 60.9 %: cytoplasmic | no hit | pfam00506/g Flu_NP Influenza virus nucleoprotein. Probab=83.28 E-value=2.9 Score=30.95 |
| **U5** | MLLIAAVTATVSYALTSVIGSLNIRKRCDGLREQMRSVDVKFYRRNNGKSVFIVWEQHQCAMWHNQKLFIYDHWHGNEVFKENMLSADTFNLRSSELVLVYDFHEHTKNLQKNFPNEYMMRELFEVNGNCEEVLLNFRASPDDKFPALVKQLYTVSDELNIIAVVYDALDKKILTC | membrane protein (1 TM) | signal peptide (1TM) | 30.4 %: mitochondrial 21.7 %: cytoplasmic | no hit | none |
| **IVSP2-1** | MAATVSLAEHQDESAVHDGRLQTLKAVQQFRHNVIGQLEAFEELFGPSNTLMPLSYSLQSKSKYEFVDFGYKLCRDHEVLIGPIYMYGLCKSTEHDKSIAKLNTDISEGKERLMMLISNCDNISHKPSGSLLSLIFNDMANKLDNVLSNAEIVKRITSRYPRFDALAFARNKKNILQKYISGKENYSCDPVRLRVDIAPTTDENYTCYEISQGLCRTKGHCYFMPPMLRRGYLILKMPYIRAVLTWKSTDYLFKLNAKMMMVEKIENTPPPSCFDISTGKAVDDTEAIFMLDGCSDVDWSDEVENVIVLPSTRELSELTTATSEHSPILPPFCYPRQDLNDNGSKSEPDTFEDDDVDMTADVSVHQGKILSCAISDFIKTIRDEGNWSPSIANLSKFVNRFIRVMKRKKASLLYTDGRTVNVNGSVVRELSITDSRTIQTPAAFEDLVEQYYDRFRLNCDTDEIMRSCWNETFSHEETTTWHSCQLTNKKKRSATSITHNASKNRQTKR | soluble protein | 2 Von Willebrand factor type A domains | 82.6 %: nuclear | no hit | none |
| **N-1** | MNGDDDDDSRGSVGLLNYSEFDGGNMLQCEDDLEEEYNDEAENGGPEIYQTVSSDDELDGKLAISQSLPVVESMSLECPSVPSLKKIVKRKPTAGGKNVLVGKVRKPDNTAPAGNAFSSEKKKSTIVSKERPAIETLSKGDSAAFAKKSSVKKKKPVLKPKDPKEESSYVPAAAAEESRNKAKVAVKKSSKLVAMAAEEQPPVKKGRRDPKHQPEGVSLPPVTKDGNSPSYRETQVQTFPQVNTSRKNSRTSGGTSTKSFLVLNSDIGDGRFWMETLAGDFASTGYNALLKNSQFTEQQAYLLQHSFYQLCRKLSDISSYSDRFMKTHKCIECDFSISHQCMSIDFSQYVPASANITGVTNIAPQSTTMCVCQFAFFHSHPTQPRVILANTLSWKSCSHMLELNRSLSLRCPRCHMNVVMKNRNNNVCSDFANWNSLEGINRRQFFKSLENRLQASKISRPHLDELYTCNRQCCQLFHRCAENALPDNTMPMRPH | soluble protein | no hit | 73.9 %: nuclear | gi|45862538|gb|AAS79017.1| NHv1.2 protein [Campoletis sonorensis ichnovirus] Score = 288 bits (736), Expect = 1e-75 | none |
| **IVSPER-2** | | | | | | |
| **U6** | MDSCHIVTLTINIQDSAFVNWAPVFESVFHAAKSHEHCLASFNSQQPVYRVLCTIIIKTTMSTYDFTILDTEVSKKMWIAGKFYGYSKFAIFNLAHSVADPLNLNSVHEICGSSAFPQTVMMEYVKKLSVTANMDYFSTAATSTFSLKQANVTISNAFAFCCEDNNIACHRSVREVAGVRLIFNGQRAQFLNKLRSLLPRAYITALLNSASNVQFFVNVSQALMDRINSECRAFVDNHKMCRELVFYCLCCMTSHLQITPAELDEANEAEPNEKQPRIQENPVPFCQLPLEVLPHNIFAEHIKSPSFLSSLLHFDIHVNCFYEHEVENIENIRSNLLVKTHSLNTMYNT | soluble protein | no hit | 78.3 %: cytoplasmic | no hit | none |
| **U10** | MDRTTIKKCFSSEVQPLAPGFEELSPDGRVVKKRHKVTTGVNLKYYEFILTLEKICSDIATVPENAYSSVIQELFRTLANYRVLICDPMANMLVDALQRLSELNIIYVLKQTELVVMNIMLPETVKNSDAIHMFAISLRLLVLNMIVCGFARRDLHNAISFSTRERSTLDYELDVQCATMHTQSNHCCLCIAENYPKRDIDVRGADLILDDVRKEFVNYSRIRTCKCVANSNLCEQCLCSLRESLYLTYIFYKFEDEKKSKSIRDKNAELRIFKAKSLAKRDARLRATAQKINLETAGTTTPPVLDKRVRLNLTKELLETTADTAVDRHRSQKIVYEDSNLQLRVRKSGAIRFKRKSANGYDEIVILGECKEPPKIAQESTPVGSLLQGKIRRGRLIERPKKVSRKFIDKLMNDLSSKAKKRSTDIPRMQRSTLCAKKPGLAQRFVKVISTALLNNERLLNESVIAELCNVVLKLRGLVLEMTRCHCEACKSTVCESLSIEDMRPIILALFLHNEILNNNFLMDICNDKMFMSLQMPRRSAYKMYFNIVDNRKSIVKDKLAEARTRIQNVISSLMLYDASKKLAKPITLCFAIRKYGAQLHDMYRLDFNGARDNPVHDLVWLKTCTKNDSALFLYKSEKDFSLTGQASLEYNTAYHTYSVTVKSQQGNTTSFSKMFDPWLSAAGTSVLRQGPAVTSRTLNRYNKMRNRPKYAAISGVVVKSVKRDFRGSGEGMNRKRRSDMNRGKEGIMCCQATVSELEATILQIFDSMLLGHLLRVYCNILSNQIECNNTDYRGFYDQFHYLNGTRPTERYESDIFVLIADRTFKATEKVLIEDTVLLRNKGPLSMKNIPRVVKYYARSTLDAFTKFEFNSTLIVLFRPTGNNYFVIQFENDRSIIVQHIAKCVLVELVCKDPLSADAPLTATERYFENADNALFSKNEVIAIRDFLNTDNTLPKTDNSYSTLMFLVGDDNDNESTLDEAIQDCERIAEVVMKVKTVAITDRCASIVFDKIFEAAWNLEPVSFTRQIDERTLACVPAHDTDVICTWTTEERTTLCTATKYALVFVGRLMVMSDYNVLDIRLLDCFRRRISVLKCSTRAGVIIPLNRVPSCEYEVASFLKNGINHVESYRYIHKNITAGRMLVHDNFAVMYTATTTALHLSLDDMPAIKWKKVKGMFHGRHSNSGFLTMSANKLLLAVYAATVIVVNTYHKEYFTLKSSLLITSGHGLSEVNFIRLKYIVRKIVFVDGYVVSKESQRYDYKKLISLMLIGEQAVLLMHQGSLNNEAILKSVGHYHRSLHVLYVYRKLIDYFTSQYSDMPMECDPTVFMLNIVSNAKKFISNFETKTVIRVPLRCK | membrane protein (2 TM) | 1 TM (130-150) | 34.8 %: cytoplasmic 26.1 %: mitochondrial 21.7 %: nuclear | no hit | none |
| **U11** | MEYKNSSMEFSDTLLDVLNFIDSYDLSTMDVINGHSSVHLDGLAMLIDEDGHEDTLHHVYSNQTDQSIGLERSKVMTYNQDQVAVQEQNVHQSTKGVFVLQTEDVTLWRSLMESSDLYKRHCSLLVHEGNSAVTLNHTAMAAMDTFIDIVLIEAVTNKSLAFLEKFVLPVKELVLHLHRCNLKLKHTFMSSIYVCKKCNGFIATDMQHTSFRMIRELKKSNQENVRMVINAVLDVFTYPSVLPWGIRENRSTVQSAVIGGKISSVYYRSLSSKDRTSFMDFLEVFVEYYRAVALEYRGFNISTAYCELQNLFSFGS | soluble protein | 1 TM (130-150) | 65.2 %: cytoplasmic | no hit | none |
| **U12** | MTSFDLSNDMLVKRRKVTSWIIIVMDNFDRKHLKHATCATLRLFDRVVTNEVPTDLLQSVAVGVMYIACKATNIDVNIDTLLYYSGGGCSIDSLLDVAPLMRVLHEEDLSITTLPTYYITDLMHLMRLEGPLQPVVRWIIHLMSNSRYLSLETTPRMTAAIALLAYNNYLSSRSFKLSAPALLRDFSFVIKTRRHKRPERRAQ | soluble protein | **Cyclin domain (19-106)** | 65.2 %: cytoplasmic | no hit | 1f5q_B Gamma herpesvirus cyclin Probab=98.20 E-value=4.4e-08 Score=59.24 Aligned_cols=149 |
| **p12-3** | MSGFLSVTPNLISAGISMYNYYHVQELAGITVNNKMTNPDLEKYKQEMETVQWASLAAAVTTSINGVLSIFNAGRHHHHHPGHIPPPPYTEHYLGDGHVH | soluble protein | 1 TM (51-71) | 52.2 %: cytoplasmic | gi|4101554|gb|AAD01200.1| p12 [Campoletis sonorensis ichnovirus] Score = 53.5 bits (127), Expect = 5e-06 | none |
| **U14** | MLAFVLPIVGNIFLNGSKKTKKIYPFLDPLPIFNCPIFYVRTLNALLSTNTAS | soluble protein | signal peptide | 60.9 %: cytoplasmic | NS | none |
| ***U14b*** | *MMVEPIMTHSYSWYGLDVHCIRRSPNPDFASMKQASGRNINIPELELNFGLSVDWSICWDYYCTRCPRISRFFRVTH* | *soluble protein* | *no hit* | *43.5 %: cytoplasmic* | *no hit* | none |
| **p12-2** | MAGPLLAASSLFTAGVSVFNYYHVYQLGEMTISSDMNEQNLATYRSQIETVQWSSLAAAITASADFVCRIIHAAPPSHHHHGHPHWHHHHPGHVHHHMTDGLIN | soluble protein | signal peptide (1TM) | 33.3 %: extracellular, including cell wall 22.2 %: nuclear | gi|4101554|gb|AAD01200.1| p12 [Campoletis sonorensis ichnovirus] 41.2 0.027 | none |
| **IVSPER-3** | | | | | | |
| **U16** | MVKSIEDFVFQGLSTKDQPSSHLFPRKCMVTRANTNGFDALDLFAASYKPQIGQYKCFQEKNGCAPEQGYPYRIVVDLDSSDEHLLQSLLYEIGKLLEKLLCQANTSDFKVMICIMRKQQTGRFHVHLLNVVTNDLTTYKNFLIMLHEKVQAVDKGAGVNYFMVFGAIKAYKLNVNPTTPAADQCYLPWKLCCAEPNEIDLNNFVDLPGFTGGSCDEYLEDIYNYFKKHFEPFSCKTLFHALSLHRRYNPDFDVVLPSCQDSLVRCAKRRANEDSDSEGKKKVRTGKSDPINRAKESFFENVLFKLPRNYYEEYDSWIGIGKIIAYVKQNYGLHLFHKFSAQSRNKYDAEKVTATYEGLLETIKVNQGEGEDEPAIRTTSALRTLLLGSNSIIDQIEHKMFYKWKGDTHAIVGAVNCCIQQMSPMLTFPHPLNANYLFAIEFSRSDGAYRISHDTFIQSFGGQRATIDAGVERHEHYERILECFIYLAIKYYNDNHRFLRMYTAHGILDSTPFKLKNHLHAKNNAEDMRSSQYSFYRRAVVQKLNKLKKRWFLAQANGATTVSRKNNKRLWSKLYAEFEFHRALQSARKCGRYPQSVLVKAVTRSGPPRTAL | soluble protein | no hit | 65.2 %: cytoplasmic | no hit | pfam08707/g PriCT_2 Primase C terminal 2 (PriCT-2). Probab=98.03 E-value=9.9e-06 Score=52.75 Aligned_cols=58 (301-359) |
| **U17** | MALTIICGLVGLSGGIYYEYRRAKNEAQSRIEKDEFDAKAREIFNTTIHGRVRDDIFNEKVYQEFVTEDRLKDLQQVVQPVN | soluble protein | signal peptide | 44.4 %: extracellular, including cell wall | no hit | none |
| **U18** | MFRDVTQTSTYSINNFHYQGSLLHCVETARLCTHALTDTDPLNDSESPTLSERPSRASALHQKLLFSSSTRFSFLELLRQCLSSV | soluble protein | no hit | 60.9 %: nuclear | NS | none |
| **U19** | MDKVGGLVKHVIRYGKGNPDPEEWVHKYYAQHLVIEVHSFIKPMFRHVKHSSHSDVDYAAFLNNLDLQVRCILAQVSVVTLDCTRLTTHLVERNKTLVVDDLFNTILQAVMSGSGGRGYYSNDNGRNEERCSTSNIHLVIANCNVTHLWQALYNCADQNSFLCENISLIDCTRVDLGLVVKLCLCGLHFRNCKFAFNKRRNIAPNALKNLTIECSSSFNDWHREAVPTMVHYFVDRIEHRPIIVYTSELAISFKSLDTKNATRTNLAVMLFNLGISLASVTAFQINIPLADAGVPPTSPSIVKDITNAFDKMMKKLTRLAVASITDVPSFTELDALTGLTLPNAANTDLAQLRAIVYNRNERLKYYNGPPRALADLSCAWSKLSEISLDFRFCDNGSIVLYTILSRCFSLKTIKLSNVCSLNYNFLNDESNLYTPVLSSLHVECHAVCVIEPLKMMDKFGLDKLYLTLVENLHDDCPALAAKQQAYSPEKIASYIGECGKGYTTLLHITVKSTVSEIDSSIERSKLVEYELVHCLFDKFIKYRKSVKAEDNQTSFVVWFDDISNELKNRKGANVKLWQHLLHHIKKRASSLPLSLNPLQTKTNMPPVADLAFAVAFDRATTAAEKNFVKQLENSRNVACKCSASENSFAYWPCSIYDLFY | soluble protein | no hit | 33.3 %: endoplasmic reticulum 22.2 %: mitochondrial 22.2 %: nuclear | no hit | pfam05894/g Podovirus_Gp16 Podovirus DNA encapsidation protein (Gp16). Probab=85.31 E-value=1.1 Score=33.08 Aligned_cols=58 (482-570) |
| **U20** | MEEIGERKKSTLHVICRRQYDQRNAETIVESAVKVHECLRQMQNPLSVPAEFRPVTNALKSMCESILANT | soluble protein | no hit | 47.8 %: nuclear 30.4 %: cytoplasmic | NS | none |
| **U21** | MLFIVPAIACSTRSFALIRCALSLVVIHDHSNIVMYAIYVFAIIDNSAFDEAATLPSRSHFLNRDVLVVCRSSTTLLDH | membrane protein (2 TM) | signal peptide | 43.5 %: cytoplasmic | no hit | PF01688/g Herpes_gI: Alphaherpesvirus glycoprotein Probab=72.69 E-value=8 Score=29.83 Aligned_cols=61 (15-76) |
| **p53-1** | MPTLTIYRPHAPAYAAAWPLRNTISGVNSFLEYNEPREDKKRAIKTSVPEPDSVKKEIDLQVNTQTEITDGRRTEADVDATEAIIDKALVTKEFKVNCNKDMQLLKIMKYPNVRHDEPSAILSHVLVKEQDSKPFRMGAIVINDASIKGNDNITQWNVLSKYPEHSQALEAALKDSTPSTVHAFRATLRYSDDFVLALIAPPIDDMTPNAHVDDLTNEDSLKYNIVVRHSKHHQAGNGTTEENVLSRYFKRFDEKLINEIRLEAPVEPVTDDSMLKRKRRDVDESHLPRESEEVDSHESFVPLGGIDDFETPIKPREPHADITLKPTIGMKRAAPQHYQQPSVRYTPILVPVKNETLRQPSAFDTFASVALPIGTALALSAGATYMLAKRPRLAE | soluble protein | no hit | 43.5 %: mitochondrial 34.8 %: nuclear 21.7 %: cytoplasmic | gi|223587707|emb|CAR31590.1| p53-like1 protein [Hyposoter didymator] Score = 790 bits (2040), Expect = 0.0 | none |
| **U24** | MESFAKMEVELYHLQVERVFSQLAERTLNWDDNEPGDVDGEPVVFCLFMIENRNQREQPAVESFSDSSINDTNSIRKSPTKDTPMVIPKKEVPSASCKAGGYYEKKTHFVPMEVRENWDDGKADEEMVSNRGIFRSVDQNEIEIIQTMAHCYPWLLGDICSVFATLPDVWQLRVEVLFILGELMFGYLSKVESLLNVNSSRAIMQHDWESCSTANQSVRMYFQLLDAVCARASKGKKCIADLIKQSLTAHGHQPVKLILKSKMMHNYLKNGIDCPGREKKFSDQPFSQLTNMIPSNCDLVTTFGTVHVWKSFVGFLQKMYDLNAESFAGYAMCPDQNDLLIVLFDDINTAQQPTADQLTSTAWNSSYARPDESRPMSDLIDSIAGQSLLQGWRTAKHTLYGNASKNYLLQRALSKAHITQSYMYGFINDITKDVTCTARLLRNLTTINGDMETIAKDLCQSVRATMVQAMPNDLLLGALNISRIAENFGINSNRSFMELLVRNEPVKMSFKGLVDVLLNAMRKNAKHTAIDEYVFLHRMSHVRSALCDFNVIANLASQTLRKE | membrane protein (1 TM) | no hit | 47.8 %: cytoplasmic | no hit | none |

**TABLE S4.** List of the protein sequences from *Hyposoter didymator* IVSPERs not found by mass spectrometry. The name and the protein sequence are indicated. Results of the bio-informatics analyses of the sequences are indicated in the right columns (see Text S1).
