# Supplementary material for: Analysis of Virion Structural Components Reveals Vestiges of the Ancestral Ichnovirus Genome
Source: PLoS Pathog. 2010 May 27;6(5):e1000923. doi: 10.1371/journal.ppat.1000923 (PMC2877734; doi:10.1371/journal.ppat.1000923)
Supplement: Table S5 — Comparative analysis of the gene families found in Hyposoter didymator IVSPERs. Protein sequences were aligned 2 by 2 using the LALIGN program (http://www.ch.embnet.org/software/LALIGN_form.html). For each alignment, the overlaps vary in size, and the percentages of identity and similarity are given. (0.06 MB DOC) [file ppat.1000923.s006.doc]

|  | **Gene product** | **size (aa)** | **LALIGN Fasta alignment results** | | |
| --- | --- | --- | --- | --- | --- |
| ***p12* gene family** | **p12-1** | 77 | 28.6% identity (61.9% similar) in 63 aa overlap |  | 20.0% identity (67.1% similar) in 70 aa overlap |
|  | **p12-2** | 104 |  | 43.0% identity (75.0% similar) in 100 aa overlap |  |
|  | **p12-3** | 100 |  |  |  |
|  |  |  |  |  |  |
| ***N* gene family** | **N-1** | 492 | 63.3% identity (82.0% similar) in 499 aa overlap |  | 44.6% identity (70.3% similar) in 74 aa overlap |
|  | **N-2** | 495 |  | 45.3% identity (66.3% similar) in 86 aa overlap |  |
|  | **N-3** | 93 |  |  |  |
|  |  |  |  |  |  |
| ***p53* gene family** | **p53-1** | 395 | 34.6% identity (63.3% similar) in 289 aa overlap |  |  |
|  | **p53-2** | 323 |  |  |  |
|  |  |  |  |  |  |
| ***IVSP1* gene family** | **IVSP1-1** | 193 | 36.0% identity (76.0% similar) in 200 aa overlap |  |  |
|  | **IVSP1-2** | 253 |  |  |  |
|  |  |  |  |  |  |
| ***IVSP2* gene family** | **IVSP2-1** | 509 | 65.7% identity (92.3% similar) in 143 aa overlap |  |  |
|  | **IVSP2-2*** | 143 |  | ***N-terminus only** |  |
|  |  |  |  |  |  |
| ***IVSP3* gene family** | **IVSP3-1** | 527 | 57.2% identity (83.4% similar) in 523 aa overlap |  |  |
|  | **IVSP3-2** | 630 |  |  |  |
|  |  |  |  |  |  |
| ***IVSP4* gene family** | **IVSP4-1** | 431 | 52.7% identity (80.1% similar) in 402 aa overlap |  |  |
|  | **IVSP4-2** | 446 |  |  |  |

**TABLE S5.** Comparative analysis of the gene families found in *Hyposoter didymator* IVSPERs. Protein sequences were aligned 2 by 2 using the LALIGN program (http://www.ch.embnet.org/software/LALIGN_form.html). For each alignment, the overlaps vary in size, and the percentages of identity and similarity are given.
