# Supplementary material for: Analysis of Virion Structural Components Reveals Vestiges of the Ancestral Ichnovirus Genome
Source: PLoS Pathog. 2010 May 27;6(5):e1000923. doi: 10.1371/journal.ppat.1000923 (PMC2877734; doi:10.1371/journal.ppat.1000923)
Supplement: Table S6 — List of the primers used for qPCR and classical PCR. For the housekeeping Hyposoter didymator genes, the identity of the Blastx match against the NCBI database is indicated on the right. (0.09 MB DOC) [file ppat.1000923.s007.doc]

| **Name** | **forw primer** | **rev primer** |  |
| --- | --- | --- | --- |
| **A- Primers for qPCR** | | |  |
| **IVSPER-1 (BQ clone)** | | |  |
| **U2** | CCCAATGCGGATACAGATGG | ATGGTCGTCATTGTTGTCGC |  |
| **p53-2** | TGAACAAAAATGCTCGCACG | GGCATTGCGACATATCCGAC |  |
| **U5** | GAAATTGCGAAGAAGTGCTGC | ACTTGTCATCGGGAGAAGCG |  |
| **IVSP2-1** | AACGGAAGTGTAGTGCGCG | TTGGATCGTCCGACTGTCAG |  |
| **N-1** | AGGTGCCACATGAACGTCG | CGAGCAAACGTTGTTGTTGC |  |
| **IVSPER-2 (BR clone)** | | |  |
| **N-3** | AGGTCACGAGCTCCACATGG | TCTGCTGACGATTCTCGAGGT |  |
| **U6** | GAACCGAATGAGAAGCAGCC | GCAGAAGGGAACGGGATTTT |  |
| **U8** | TGGCTGGCTCAAAAGGAATC | TCATTTGTCGGCTTTCAGTCG |  |
| **U10** | GGACGATGTACTTGAGCCGG | GGACGATGTACTTGAGCCGG |  |
| **U11** | CGGATTCATCGCAACTGACA | TCCCGAATCATTCGAAAGCTT |  |
| **U12** | CAGTCGCTACTTGAGCCTCGA | GCAATCGCCGCAGTCATAC |  |
| **p12-3** | AGCAGGAAATGGAGACGGTTC | TGGTTACTGCCGCTGCTAAAG |  |
| **U14** | GGTGGCTTCTCTGACGGTGA | TGACCCATCTGCTTCGTCG |  |
| **p12-2** | TCTAGCAGCGGCAATAACCG | TGGATAATGCGGCACACAAA |  |
| **IVSP2-2** | GAAGGCAAGGAACGACTGATG | CGCCATGTTGTCACAGTTCG |  |
| **IVSPER-3 (BT clone)** | | |  |
| **U16** | ATTGCTGTTGCTTTCCACGAG | ACACAGAAGATTGCAGTCGCA |  |
| **U17** | TTTAGACGGTATGGCGGCTC | GAACTGTTTTCGACATTCGCTG |  |
| **U18** | TCCAACGTTATCGGAGCGAC | AATTTTTGGTGTAGCGCGGA |  |
| **p12-1** | GCGGAAACCATCGTAGAATCA | AATCCCCGAGACATTGGTGA |  |
| **U19** | AAAACCAATATGCCCCCTGTC | GTCGAATGCAACGGCAAAG |  |
| **U20** | GTGATATGCAGGCGCCAATA | TTCTACGATGGTTTCCGCATT |  |
| **U21** | TGCAAATGATCGAGTCGAGC | TCCATGCTTTTCATTGTCCCT |  |
| **p53-1** | ACCACTGGGAGGAATCGATG | GGTTCTCGTGGCTTTATGGG |  |
| **U24** | AGAGCGTGCGTGCAACAAT | CGAGCAGCAAATCGTTTGG |  |
| **N-2** | CCATCGTTGCGCTGAGAAC | GGTCGCATGGAAACGGTATT |  |
| ***Hyposoter didymator* genes** | | | **Blast x results:** |
| **L55** | TCAACGTGGATTATTGCGAGC | TGTCCAAAGGCAGAGCAATG | gi|21356717|ref|NP_650780.1| mitochondrial ribosomal protein L55 CG14283-PA [Drosophila melanogaster] |
| **CytC** | GCGAGCTTCTCACGATCATG | TCGAATGGCAAATTGCCAC | gi|157129539|ref|XP_001661715.1| cytochrome c oxidase, subunit VIIC, putative [Aedes aegypti] |
| **HIS1** | CGCCGATATGGTTACATCCG | GAGCCATTGCGTTCCTTGAG | gi|110749616|ref|XP_001121111.1| PREDICTED: similar to Histone H1 [Apis mellifera] |
| **ELF1** | ACTCAAGTGATGGCACGAAGAA | TGTAAATAGCTCGCGTATTTTGG | gi|156546950|ref|XP_001605623.1| PREDICTED: similar to elongation factor 1-alpha [Nasonia vitripennis] |
| **XRCC1** | GCCCCAATAAGGATTCTGGC | TCGTCTCCTGATCCGCTACC | gi|156554771|ref|XP_001605823.1| PREDICTED: similar to DNA repair protein XRCC1 [Nasonia vitripennis] |
| **HdIV genes** | | |  |
| **SH-BQ N** | TACATCCAGGCTTGGTGAACC | TTTTAGGCGCGATGAAAGTTG |  |
| **Vinnexin** | GCCATCATCAGTGGAGTCGTT | CGACGCTAGCAGAGCAATCC |  |
| **B- Primers for PCR** | | |  |
| **IVSPER-1 (BQ clone)** | | |  |
| **p53-2** | TGCATTGCTGACTTCTTTGG | CATGCTTGTTTTGCTGCACT |  |
| **N-1** | CTTCTTCCACTCCCATCCAA | GAAGCCTGCAGTCGATTCTC |  |
| **HdIV gene** | | |  |
| **SH-BQ N** | AGGCTGGCTCAAACTTGAAA | GTAATGCTTCGGCCTGTGAT |  |
| **IVSPER-2 (BR clone)** | | |  |
| **U8** | TGGCCGTAAAAGTTGAATCC | GTCGACGGAGATCGATGAAC |  |
| **U9** | TTCCTCGTCGGTTTTATTGC | TCCATGATCGTCAACCTCAA |  |
| **U11** | GTTGCTTTCACCCAACTCGT | CCATCGAGGTGGACAGAACT |  |
| **U13** | CCAAGAACGCCAGTCAAAAT | GAGAAGCCATCAGGCAAGAC |  |
| **p12-3** | CAAGCGGAAAGCTAGCAGAT | AGGATCGAGGAAGGGGTAAA |  |
| **U14** | GTCTACCAACACCGCCAGTT | ATTGACCCATCTGCTTCGTC |  |
| **p12-2** | CACTGCTGGCGTATCAGTGT | CTCTCGCTGCTCTCGAAAAT |  |
| **IVSP4-1** | GCAGAAAGCCTCTTTTGACG | GGCAATGTAGACAGCAAGCA |  |
| **IVSPER-3 (BT clone)** | | |  |
| **p12-1** | GTTACTTGTCCCTCGGTTCG | CCACCACTCACAAGACTGGA |  |
| **p53-1** | CGAACGAGGATTCGCTAAAG | CGTGCGAATCAACTTCTTCA |  |
| **N-2** | AGTTCGGCTTCTTCCACTCA | GAAGTTTGCAGCCGATTCTC |  |
| ***Hyposoter didymator* gene** | | |  |
| **ELF1** | TCGATCGTTCGATAGCAGTG | TGTAAATAGCTCGCGTATTTTGG | for control : cDNA synthesis and DNA contamination of RNA samples |

**TABLE S6.** List of the primers used for qPCR and classical PCR. For the housekeeping *Hyposoter didymator* genes, the identity of the Blastx match against the NCBI database is indicated on the right.
